# Additive Manufacturing Engineering for Electrochemistry: Conductive High‐Density Polyethylene Filament Expands the Materials Landscape

**DOI:** 10.1002/smsc.70425

**Published:** 2026-10-08

**Authors:** Muhzamil A. Khan, Elena Bernalte, Ridho Asra, Robert S. Tapping, Ana C. M. Oliveira, Alan M. Jones, Rodrigo A. A. Muñoz, Samuel Rowley‐Neale, Robert D. Crapnell, Craig E. Banks

**Affiliations:** ^1^ Faculty of Science and Engineering Manchester Metropolitan University Manchester Great Britain; ^2^ School of Pharmacy School of Health Sciences College of Medicine and Health University of Birmingham Edgbaston Great Britain; ^3^ Institute of Chemistry Federal University of Uberlândia Uberlândia Minas Gerais Brazil

**Keywords:** 3D printing, additive manufacturing, carbon black, composites, electrochemistry, electrosynthesis, high‐density poly(ethylene)

## Abstract

Additive manufactured electrochemical platforms produced using bespoke conductive filaments are becoming increasingly established within modern electrochemical research; however, the latest developments within the field of additive manufacturing electrochemistry using fused filament fabrication technology have shown that it is constrained by the available material pool. Here, we introduce the first conductive high‐density poly(ethylene) (HDPE) filament engineered for additive manufacturing implementation as a new alternative for conductive, chemically resilient filaments which is paramount to unlocking new applications. Using a thermal compounding process that avoids environmentally harmful chemical solvents, 40 wt% carbon black (CB) was incorporated into HDPE and extruded into a flexible, plasticizer‐free filament with a low bulk resistivity of 16.4 (±1.0) Ω cm^‒^^1^. Following printing, electrochemical activation was required to expose the conductive carbon black phase and generate the functional electrode surface, particularly for inner‐sphere electron transfer. Optimized additive manufacturing produced electrochemical platforms with reproducible performance and heterogeneous electron transfer kinetics corresponding to 2.93 (±0.19) × 10^−3^ cm s^−1^. Electrochemical stability testing revealed that the CB/HDPE additive manufactured electrodes exhibited consistent voltammetric responses for over 60 scans, representing an improvement compared to other conductive polymer feedstocks. Over a 70‐day period, the CB/HDPE electrodes also maintained chemical stability following immersion in six different solvents. The material’s versatility was demonstrated through proof of concept for the electrosynthetic oxidation of chlorpromazine‐to‐chlorpromazine sulfoxide and the electroanalytical determination of caffeine in commercial energy and coffee beverages, which was validated against liquid chromatography mass spectroscopy, both in aqueous and organic media. This work establishes conductive HDPE as a new entrant to the additive manufacturing electrochemistry toolkit, expanding the material landscape and enabling applications previously inaccessible with existing polymers.

## Graphical abstract

Conductive high‐density poly(ethylene) (HDPE) is introduced as a new material platform for additive manufacturing electrochemistry, expanding the limited polymer feedstocks currently available. A solvent‐free thermal compounding process incorporating 40 wt% carbon black produced a flexible, plasticizer‐free filament with a low bulk resistivity of 16.4 (±1.0) Ω cm_‐1_. Following electrochemical activation, additive manufactured CB/HDPE electrodes exhibited reproducible electrochemical performance and excellent chemical stability across a range of aqueous and organic environments. The versatility of the platform was demonstrated through electrosynthetic oxidation of chlorpromazine and electroanalytical determination of caffeine in commercial beverages, validated against liquid chromatography mass spectroscopy. Conductive HDPE therefore broadens the materials landscape for additive manufacturing electrochemistry and enables applications across electroanalysis and electrosynthesis.

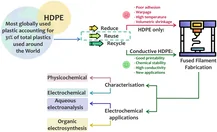

## 1 Introduction

Additive manufacturing has revolutionized the way complex components are designed and fabricated, offering unparalleled freedom in geometry, rapid prototyping, and cost‐effective customization [1, 2]. Among its techniques, fused filament fabrication (FFF) stands out for its accessibility and scalability, enabling the creation of intricate structures with minimal material waste. Despite this promise, the progress of FFF in functional applications, particularly electrochemistry, has been hindered by a limited pool of printable, conductive, and chemically robust polymers [3]. Expanding this material landscape is critical to unlocking new capabilities and broadening the scope of additive manufactured electrochemical devices.

To address this need for new functional materials, we turn to the thermoplastic polymers used as the base of filament production. Plastics are among the most widely used materials in the world, serving as essential components across industries ranging from packaging and construction to health care, automotive, and electronics due to their versatility, durability, and cost‐effectiveness. According to the United Nations, approximately 430 million metric tonnes of plastic are produced every year globally, highlighting their importance [4]. While a vast number of plastics are available, one of the most widely used is poly(ethylene) (PE), which contributes to more than 30% of all plastics used worldwide [5]. One variant of PE is high‐density poly(ethylene) (HDPE), which is synthesized through the polymerization of ethylene in the presence of a Ziegler Natta catalyst under low temperature and pressure [6]. HDPE is comprised of linear hydrocarbon chains with minimal branching, allowing for a tightly packed crystalline structure. By nature, the structure of HDPE is directly correlated to the observed properties; for example, it exhibits excellent chemical resistance toward acids, bases, and certain organic solvents. This can be attributed to the high crystallinity and non‐polar backbone of the polymer [7, 8]. Additionally, the close packing of the polymer chains results in a high material density, contributing to HDPE’s rigidity, strength, and durability [9]. Furthermore, strong intermolecular forces give HDPE a relatively high melting point and a good thermal stability. Its resistance to moisture and UV radiation also enhances its suitability for outdoor applications and long‐term use. These favorable properties explain the extensive use of HDPE in diverse applications such as piping systems in construction, fuel tanks in automotive manufacturing, and containers and packaging for consumer goods [7, 10].

HDPE has recently been implemented in FFF additive manufacturing, representing a significant advancement due to the material’s processing challenges. HDPE exhibits poor adhesion, bonding effectively only to heated HDPE substrates, and requires higher processing temperatures compared to conventional thermoplastic filaments. Furthermore, HDPE is highly susceptible to warpage and volumetric shrinkage during cooling, which further complicates its reliable use in FFF processes. However, work completed by Schirmeister et al. [11] demonstrated how for the first time HDPE could be additive manufactured using FFF technology while also maintaining mechanical properties of that of injection molded HDPE. Furthermore, Spoerk and Sapkota found how through the use of fillers, the effect of warpage can be minimized drastically [12]. Through these works, the FFF additive manufacturing of HDPE was made possible. The stabilization of HDPE through the use of fillers opens new research opportunities. Previous studies have shown that carbon black (CB) can stabilize polymers such as polypropylene while imparting electrical conductivity [13]. The development of a conductive HDPE filament compatible with FFF therefore enables emerging research areas, including additive manufacturing for electrochemistry, to utilize these thermoplastics.

Electrochemistry research has seen the adoption of FFF additive manufacturing engineering in recent years due to the attractive advantages previously mentioned. By opting to leverage additive manufacturing, researchers are able to significantly reduce costs by using commercial filaments such as Protopasta to develop working electrodes [14, 15]. Traditional working electrodes can cost upwards of £100, whereas simple additive manufactured electrodes tend to use less than 1 g of material and Protopasta typically costs £50–100 for a 500 g spool [3]. Poly(lactic acid) (PLA) makes up the bulk of the filament, with carbon black acting as the conductive filler. However, commercial filaments have shown limited conductivity and performance, presenting significant challenges for their use in practical experiments, thereby constraining the advancement of additive manufacturing in electrochemical applications [16]. There are, of course, ways of improving the overall performance of additive manufactured electrodes using commercial filaments, such as through applying different surface treatments, including chronoamperometry in alkaline medium [17, 18, 19, 20]. This widely applied methodology looks to relieve additive manufactured electrodes of their limitations, including high background currents and poor electron transfer kinetics due to the insulating surface layer of polymer formed during the printing process. Through activation, such surface polymer can be stripped away, exposing the conductive filler and increasing the electrochemical transfer rate. Although improvements have been seen, the intrinsic material properties of PLA, such as poor chemical stability [21] and solution ingress [22, 23, 24], are still major drawbacks in its application to real‐world scenarios.

To overcome these limitations, researchers have expanded toward investigating alternative thermoplastics, which show potential for improving on these severe limitations. For example, conductive recycled poly(ethylene terephthalate glycol) (rPETg) [25], the inclusion of metal nanoparticles which have been used to functionalize conductive materials and open new fields of application, e.g., hydrogen production in green energy [26, 27], a conductive recycled polypropylene (PP) [28], and thermoplastic polyurethane (TPU) [29] have been reported. These advancements in additive manufacturing engineering electrochemistry provided researchers with the ability to explore novel applications; however, the material pool available is still small, limiting true innovation.

In this work, we report, for the first time, the development of a conductive carbon black/high‐density polyethylene (CB/HDPE) filament for additive manufactured electrochemical platforms (AMEP). The scientific objective is not simply to demonstrate filament extrusion but to determine whether HDPE can provide a chemically resistant polymer matrix while retaining the conductivity, electrochemical accessibility, and electron transfer performance required for a functional electrode. The novelty therefore lies in the introduction and systematic evaluation of HDPE as a new conductive polymer matrix rather than in the development of a new FFF process or electrode architecture. Its physicochemical and electrochemical performance is critically compared with previously reported CB/PLA, CB/PP, and CB/TPU systems through material characterization, electrochemical benchmarking, prolonged immersion testing, and proof‐of‐concept electroanalytical and electrosynthetic applications.

## 2 Results and Discussion

### 2.1 Filament Production and Physicochemical Characterization

The fabrication of the conductive composite for filament production was successfully achieved using thermal mixing, a technique chosen for its ability to generate high shear forces during processing. The experimental procedures are provided in the Experimental Section of the Supporting Information. These high shear forces ensured a uniform dispersion of carbon black (<20 nm, CB) throughout the high‐density polyethylene (HDPE) matrix, a critical factor for achieving consistent low electrical resistance and further reproducible electrochemical performance. The optimized composite formulation comprised 60:40 wt% HDPE:CB, determined through preliminary trials that identified this ratio as the maximum achievable CB loading without compromising the composite integrity. Attempts to exceed the 40 wt% threshold of CB led to incomplete incorporation of CB, confirming the importance of this balance for structural and functional stability. After granulation and extrusion at 210 °C, the first CB/HDPE conductive filament was produced, introducing a novel polymer composite specifically tailored for additive manufacturing in electrochemical applications. Figure 1A provides visual confirmation of the successful production of a flexible and robust conductive filament, with an average diameter of 1.71 (±0.03) mm, suitable for FFF additive manufacturing. Note that this filament does not have any plasticizers since this combination of CB with HDPE allows for flexible filament and printable filament. To understand the conductive properties of the CB/HDPE filament, bulk resistivity was calculated across 10 cm ranges, providing a value of 16.4 (±1.0) Ω cm^−1^. With no previous reports of conductive HDPE, benchmarking was completed against other conductive CB/polymer filaments reported in the literature; among these filaments, the commercial filament, Protopasta (CB/PLA), demonstrated a bulk resistivity of 228 (±17) Ω cm^‒^^1^, while bespoke conductive PLA, PP, and TPU filaments displayed bulk resistivities of 86.4 (±5.4), 24.7 (±3.0), and 31 (±4) Ω cm^‒^^1^, respectively [28, 30, 31]. This demonstrates the significantly low resistivity of the new CB/HDPE filament compared to other alternatives.

**FIGURE 1:**
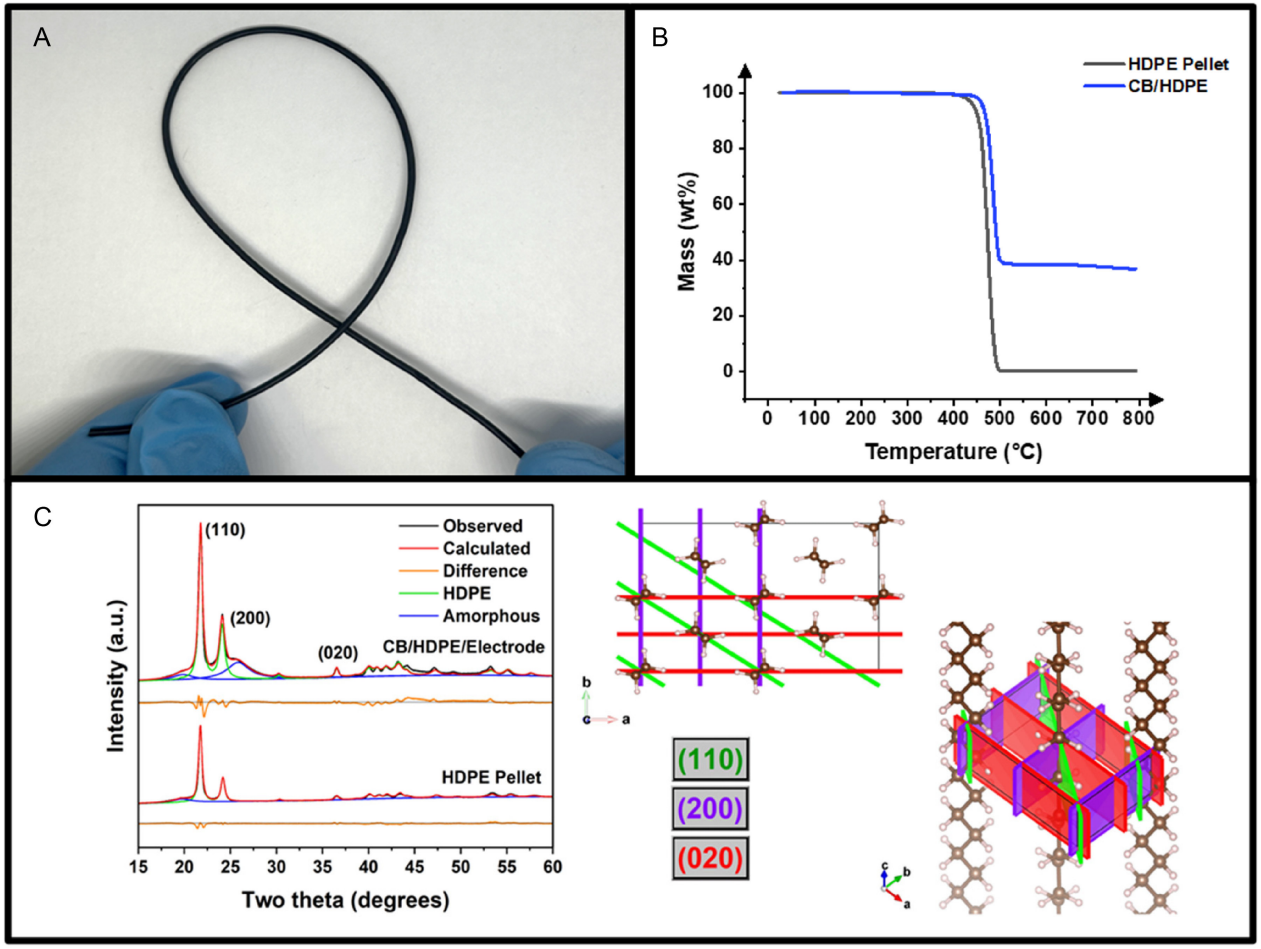
(A) Image of the CB/HDPE filament displaying its flexibility. (B) TGA of the HDPE pellets (black) and CB/HDPE filament (blue). (C) Rietveld plot of HDPE pellet and additively manufactured CB/HDPE (left) and a projection of the HDPE lattice showing the different miller planes (right).

To establish the thermal characteristics of the filament and understand how the production method impacted the material properties, thermogravimetric analysis (TGA) was conducted in triplicate on the HDPE pellets as well as the CB/HDPE filament produced. The resulting data from TGA analysis are shown in Figure 1B, where it is evident that upon the addition of CB to the polymer, the onset of thermal degradation temperature increases. For the HDPE pellets, the onset degradation temperature was 433 (±2) °C, which aligns with data seen in the literature and increases up to 455 (±1) °C for the CB/HDPE filament [32]. This increase in onset degradation indicates that the introduction of CB into the filaments provides thermal stabilization and slows the rate of decomposition. Through analyzing the residual plateau after HDPE degradation, the precise carbon black (CB) loading within the filament can be determined. Subtracting the plateau value of the neat HDPE pellets from that of the CB/HDPE composite yields a CB content of 39 (±1) wt%, indicating that no significant loss of conductive filler occurred during processing.

AMEP are fabricated as described in the Experimental Section. This design was applied for the electrodes to allow for direct comparison toward other additive manufactured electrodes seen in the literature. The printing of the CB/HDPE filament was completed using a 210 °C nozzle temperature and a 100 °C bed temperature. To allow for the filament to adhere to the build plate, polyethylene tape was applied prior to bed heating. It is important to highlight that without the use of polyethylene tape, the molten filament would not be able to firmly stick as traditional build plates are not viable for accommodating HDPE. A video demonstrating the printing of the CB/HDPE electrodes can be found in the supporting media, where the filament shows good printability, strong layer‐to‐layer adhesion, and a high‐quality finish. Figure S1 shows the produced electrode and outlines the respective dimensions.

X‐ray diffraction (XRD) analysis is employed to provide insights into the changes of HDPE upon being subject to different steps of the manufacturing process. HDPE crystallizes in the orthorhombic *Pnma* lattice with nearly all‐*trans* chain conformations, giving rise to the characteristic reflections at approximately 21.6° and 24.0° 2*θ* (Cu K*α*), indexed as (110) and (200), respectively [33]. The structure of HDPE is well known and has been studied through various techniques such as neutron and XRD. Therefore, for the purpose of refinement, it was only deemed necessary to perform whole powder pattern fitting (WPPF, LeBail method). Under thermal and pressurized stress, HDPE has shown to change the space group from orthorhombic to monoclinic [34], although no such features are observed in our XRD patterns. Figure 1C presents the WPPF plot of HDPE pellets and the CB/HDPE AMEP accompanied by an image of the crystal structure of HDPE with observable (110) and (200) reflections. Additionally, the plots for scans taken throughout the electrode production process are provided in Figure S2A–F. The HDPE pellets exhibit lattice parameters of *a* = 7.366 Å, *b* = 4.921 Å, and *c* = 2.529 Å. The corresponding coherence lengths were *L*_(110)_ = 280 Å and *L*_(200)_ = 222 Å [33]. Differential scanning calorimetry (DSC) analysis of the HDPE pellets yielded a heat of fusion of 197.12 J g^−1^ that corresponds to a crystallinity of 67.3% (Figure S3). Following thermal mixing, neat HDPE retains the orthorhombic crystal structure but shows a slight expansion of the unit cell to *a* = 7.408 Å, *b* = 4.934 Å, and *c* = 2.544 Å. At the same time, the coherence lengths decrease marginally to *L*_(110)_ = 261 Å and *L*_(200)_ = 217 Å.

Mechanical grinding introduces a microstructural response. The lattice parameters remain mostly consistent at *a* = 7.385 Å, *b* = 4.933 Å, and *c* = 2.537 Å, whereas coherence lengths decrease to *L*_(110)_ = 193 Å and *L*_(200)_ = 202 Å, indicating a pronounced loss of directional order. Similarly to the thermal mixing process, the additively manufactured CB/HDPE electrodes show an expansion in all unit cell dimensions with *a* = 7.417 Å, *b* = 4.939 Å, and *c* = 2.546 Å. The coherence lengths increase from the grinded HDPE to *L*_(110)_ = 288 Å and *L*_(200)_ = 224 Å. The printing process appears to improve the directional order while expanding the unit cell. Furthermore, in each step, the orthorhombic structure is maintained. The DSC measurement of the additively manufactured non‐conductive HDPE/electrode supports this, for which the heat of fusion was 202.7 J g^−1^ which corresponds to a crystallinity of 69.2% (Figure S2). Incorporating 40 wt% CB preserves the orthorhombic symmetry while producing a further expansion in lattice dimensions to *a* = 7.436, *b* = 4.940, and *c* = 2.549. Remarkably, both coherence lengths decrease substantially to *L*_(110)_ = 195 Å and *L*_(200)_ = 117 Å. The DSC measurements for CB/HDPE/electrode show a heat of fusion of 95.65 J g^−1^, corresponding to a crystallinity of 54.4%, which is significantly lower than that of the CB‐free electrode. This reduction suggests that the presence of CB disrupts the development of extended crystalline lamellae, likely by impeding chain folding and crystal growth during solidification. Overall, the polymer retains its structural properties following carbon black incorporation, while electrical conductivity is simultaneously attained.

Scanning electron microscopy (SEM) analysis of the filament surface and cross section was performed (Figure 2A,B), where through a comparison of the images, it can be observed that a dense polymer matrix with embedded CB domains exists on the surface of the CB/HDPE filament, while it is evident that a substantial amount of the conductive filler resides toward the center of the filament in a more porous domain. This indicates an expected “gloving” effect upon extrusion, where the higher levels of HDPE migrate to the surface of the filament, a common phenomenon seen with highly loaded filament made from other thermoplastics. To improve electrochemical performance, removal of this surface layer of insulating material will become crucial, and therefore, it is expected that a post‐print or “activation” procedure will be required. As such, the additively manufactured electrodes were activated using the most common method reported within the literature involving applying chronoamperometry in 0.5 M NaOH [35] and analyzed using SEM (Figure 2C and D). One can see that there are similarities to the filament surface, with a large amount of the polymer coating, but clear indications of the presence of carbon black embedded beneath. However, when compared to the electrodes post‐activation, significant amounts of conductive filler are exposed. This porous network facilitates the access of redox active molecules to the surface of the conductive filler, crucial for the electrochemical performance of the printed parts, especially toward inner‐sphere redox probes [36].

**FIGURE 2:**
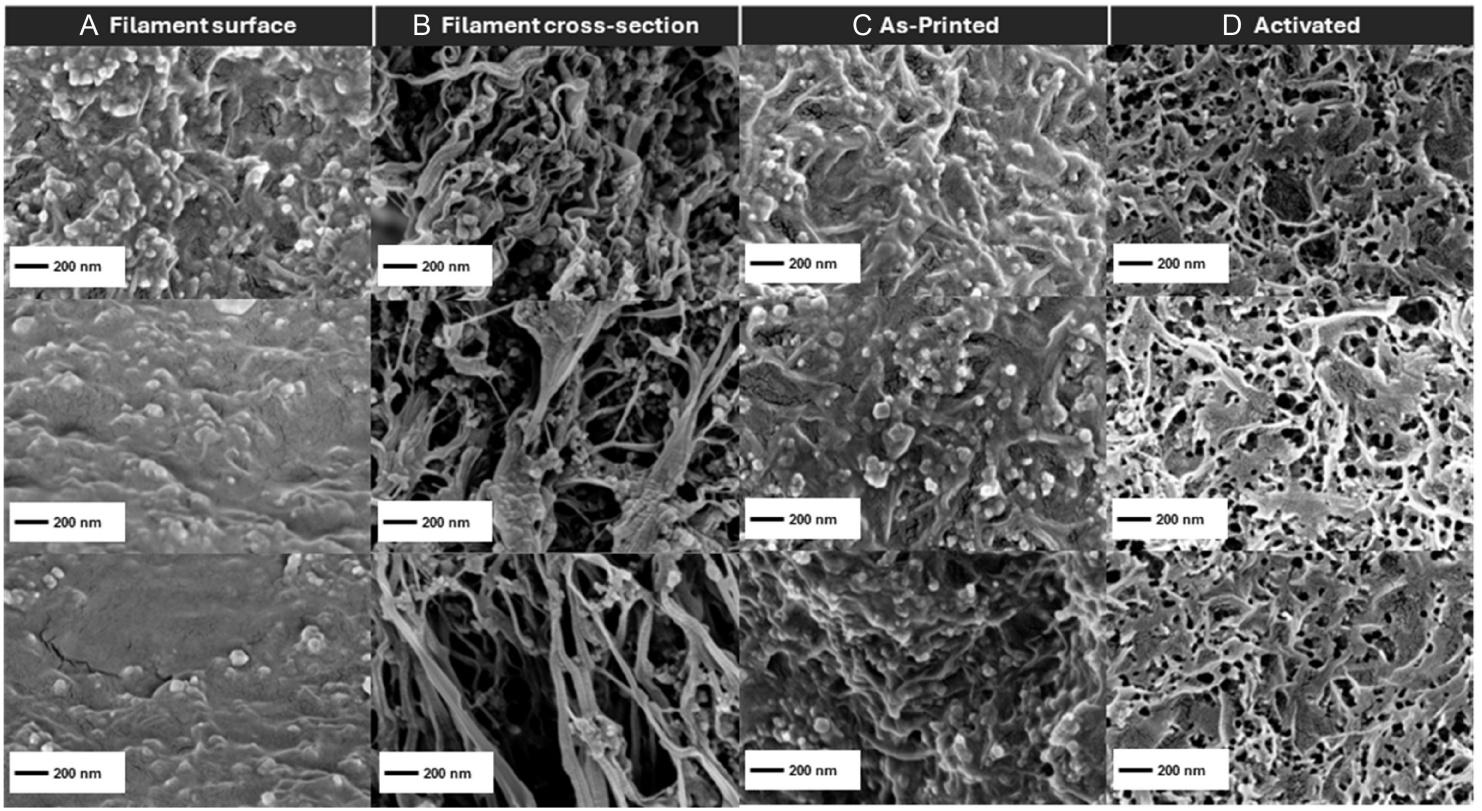
Shows SEM images for the (A) filament surface, (B) filament cross section, (C) as‐printed CB/HDPE AMEP, and (D) activated CB/HDPE AMEP.

Raman mapping of the peak height ratio of the D/G peaks for the as‐printed and activated is shown in Figure S4. Analysis of the maps revealed that following the activation procedure, an increase in high‐ratio (red) regions occurred. This indicated that the activation of the electrodes introduces edge plane‐like/defect sites, which are known to increase electrochemical activity. Raman spectroscopy was next applied to both the CB/HDPE filament and additively manufactured electrodes. Figure 3A shows the comparison of the data for the surface and cross section of the filament as well as the as‐printed and activated additively manufactured electrodes. Peaks at 1338, 1572, and 2680 cm^−1^ were observed and assigned to the D‐, G‐, and 2D bands, respectively [37]. By looking at the ratio of the D/G peaks, an understanding of the order/disorder of the material can be found. Both parts of the filaments and the as‐printed and activated electrode displayed similar values in D/G peaks, the filament surface and cross section have a value of 1.07 and 1.08, respectively, and the as‐printed and activated electrodes both had a D/G ratio of 1.09. These values align with that seen in the literature, as CB is inherently disordered, unlike fillers such as graphite. X‐ray photoelectron spectroscopy (XPS) analysis was conducted on the as‐printed and activated CB/HDPE additively manufactured electrodes. When fitting the C 1s spectra for the as‐printed and activated HDPE (Figure 3B,C), four symmetric peaks were required for an appropriate fit: a major peak corresponding to C─C (sp^3^) bonding at 285 eV and three minor peaks attributed to C─O, C═O, and O─C═O. In addition, an asymmetric peak was required at 284.5 eV, consistent with the emission from sp^2^ hybridized carbon [38, 39]. The as‐printed additively manufactured electrodes demonstrated an atomic concentration of 1.05 (±0.75)% for the graphitic peak, which increased significantly to 36.07 (±12.52)% for the activated electrode. This also supports the SEM images shown in Figure 2, where increased accessibility of the conductive carbon black after activation was observed. The atomic concentrations of the functional groups are available in Table S1, where it can be determined that upon activation, there is a reduction in the concentration for C─O, C═O, and O─C═O functional groups. Figure S5 presents the survey and O 1s spectra, which confirm the presence of carbon and oxygen. It has previously been shown through XPS analysis that HDPE inherently exhibits C/O functional groups, despite its molecular structure containing none [40]. These functional groups reside toward the surface of the material and are a result of surface oxidation due to being exposed to air [40]. Therefore, the decrease in these functional groups further confirms that the activation process is successfully stripping away surface polymer, allowing for the exposure of CB, consistent with the obtained SEM images.

**FIGURE 3:**
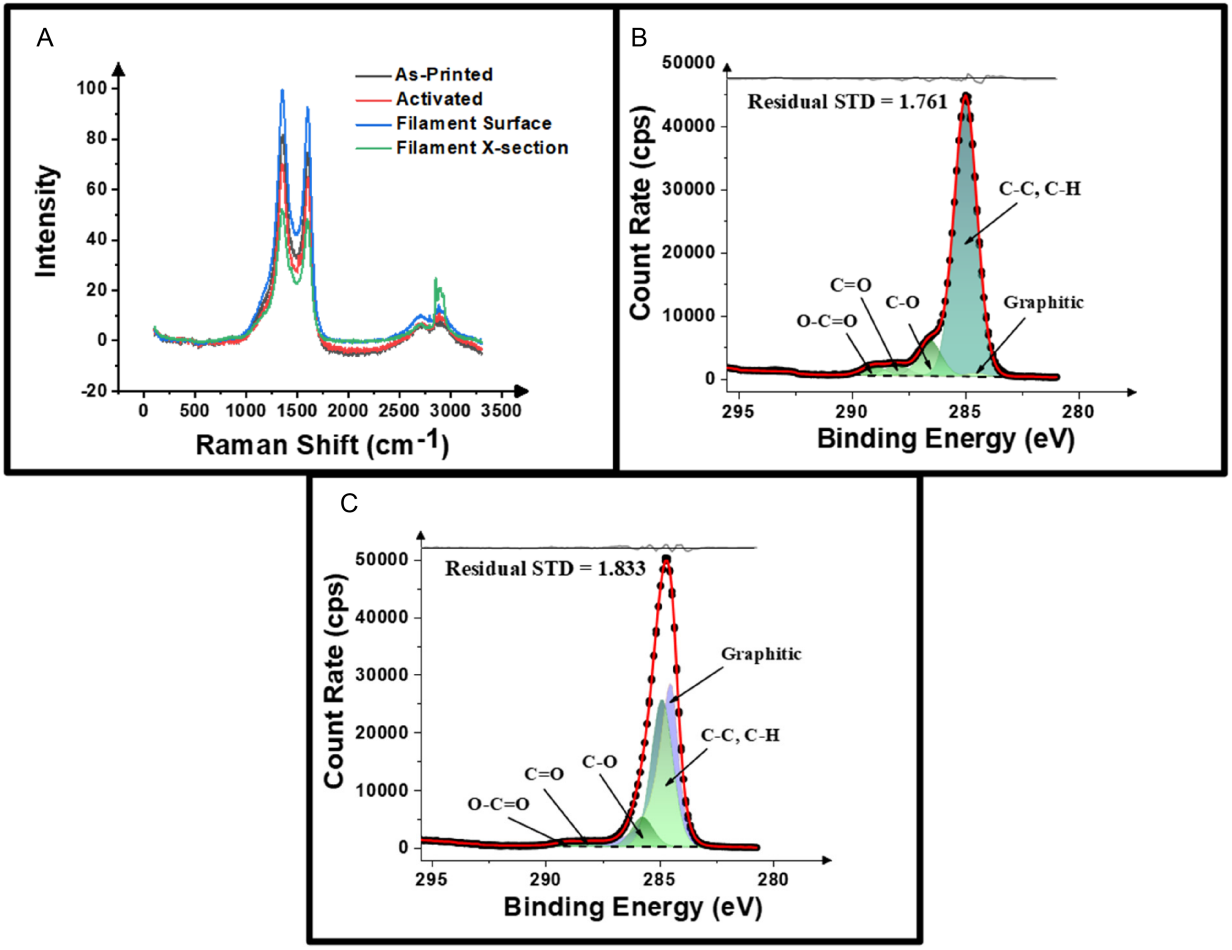
(A) Raman spectroscopy data for the additively manufactured CB/HDPE AMEP both as‐printed and activated and the filament surface and cross section. XPS C1s region for the (B) as‐printed and (C) activated CB/HDPE AMEP.

Finally, contact angle measurements were performed in triplicate on non‐conductive, as‐printed CB/HDPE AMEP, and activated CB/HDPE AMEP (Figure S6) to assess the influence of CB incorporation and the activation process on the hydrophobicity of the polymer. The resulting data showcased that throughout the production of the electrodes and post‐activation, the hydrophobicity of the polymer does not significantly change. The contact angles for the non‐conductive, as‐printed CB/HDPE AMEP, and activated CB/HDPE AMEP were calculated to be 90 (±2)°, 89 (±2)°, and 85 (±2)°, respectively. These results are expected as the C/O functional groups remain at low constant percentages following activation, as revealed by XPS, meaning that the surface polarity of the HDPE electrode was not altered and therefore hydrophobicity did not change. This consistency in hydrophobic nature is particularly interesting as when PLA is used a base polymer, it is often seen that a drastic reduction in hydrophobicity occurs following the activation process [41].

### 2.2 Electrochemical Characterization

Before electrochemical characterization, the as‐printed CB/HDPE AMEP were converted into functional electrochemical platforms through a post‐print activation step. The electrodes were activated in 0.5 M NaOH by applying +1.4 V (vs. Ag/AgCl) for 200 s, followed by −1.0 V for 200 s. As demonstrated in the SEM analysis, this treatment removes or disrupts the insulating polymer‐rich surface layer and exposes the underlying conductive carbon black phase. Unless explicitly described as an as‐printed control, all subsequent electrochemical characterization and application studies were conducted using activated CB/HDPE AMEP. Figure 4 establishes whether the printed CB/HDPE composite can function as an electrochemical platform and determines how post‐print activation influences its interfacial properties. The accessible potential window and non‐Faradaic background are first evaluated, followed by outer‐ and inner‐sphere electron transfer measurements and impedance analysis. Together, these experiments distinguish the bulk conductivity of the printed composite from the accessibility and surface chemistry of the conductive carbon phase.

**FIGURE 4:**
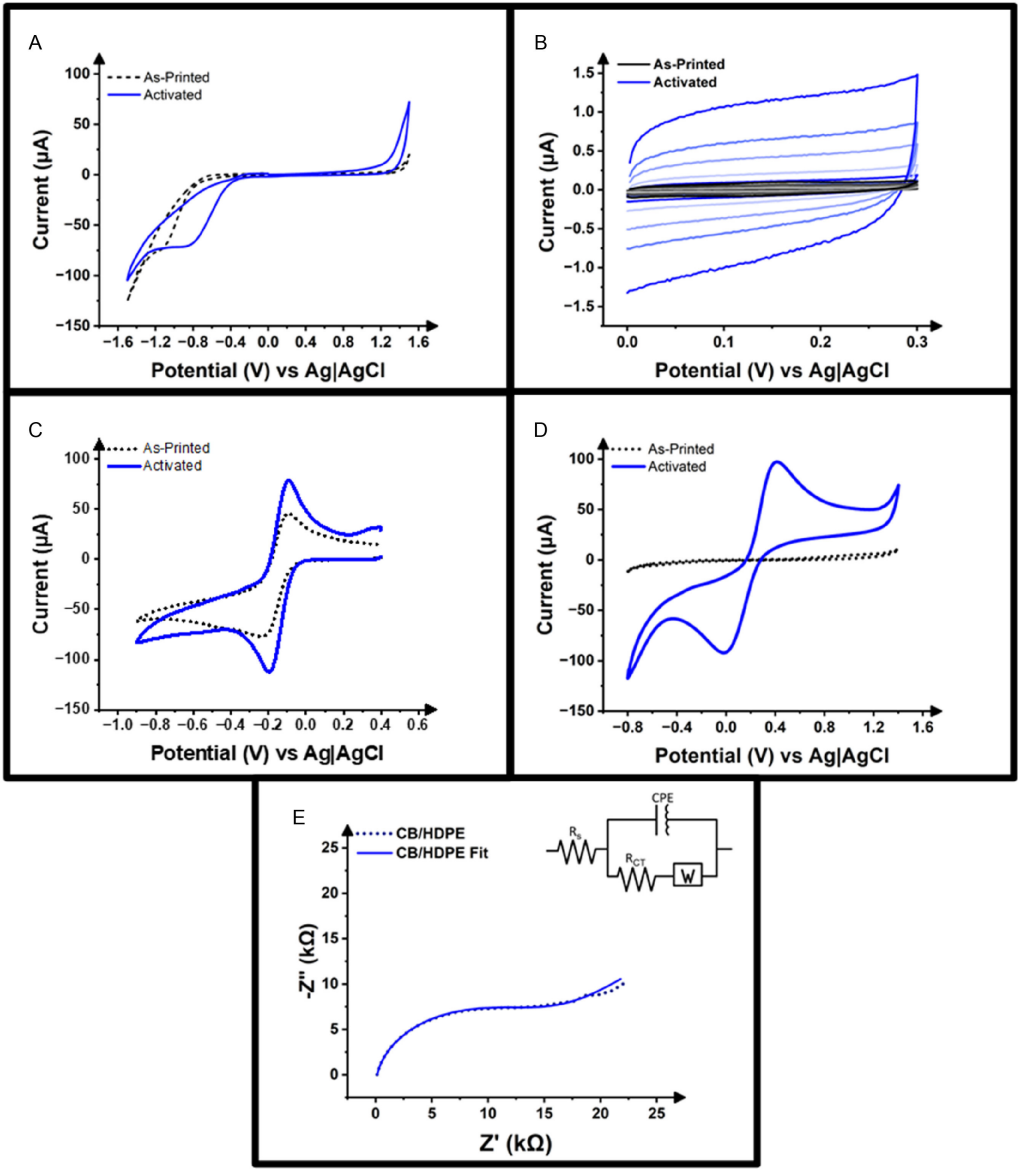
Electrochemical characterization of the as‐printed and activated CB/HDPE AMEP. (A) Accessible potential windows recorded in 0.1 M KCl at 0.05 V s^−1^. (B) Non‐Faradaic cyclic voltammograms recorded at increasing scan rates for determination of *C*_dl_. Comparison of the as‐printed and activated electrodes using (C) the outer‐sphere (1 mM [Ru(NH_3_)_6_]^3+^/0.1 M KCl)) probe and (D) the inner‐sphere (1 mM [Fe(CN)_6_]^4−^/0.1 M KCl) probe. (E) Nyquist plot and equivalent circuit fit for the activated CB/HDPE electrode in 1 mM [Fe(CN)_6_]^4−^/0.1 M KCl.

First, the additively manufactured electrodes were subjected to electrochemical characterization and benchmarking. Initially, the usable potential window was evaluated. Both as‐printed and activated CB/HDPE AMEP were analyzed via cyclic voltammetry in 0.1 M KCl over a potential range between −1.6 and +1.6 V, at a scan rate of 0.05 V s^−1^. The resulting cyclic voltammograms (Figure 4A) demonstrate a potential window between −0.8 and +1.3 V (vs. Ag/AgCl) for both the as‐printed and activated electrodes, indicating that the activation process does not constrain their operational range. Subsequently, the double‐layer capacitance (*C*_dl_) was determined by applying cyclic voltammetry at increasing scan rates within a non‐Faradaic region (0.00 to 0.30 V). The calculated capacitances (Figure 4B) were 0.196 (±0.038) and 1.914 (±0.115) μF, for the as‐printed and activated AMEP, respectively, showing that the activation results in a significant increase of the capacitance of the electrode. This enhancement is attributed to an increase in electrochemically accessible surface area and greater exposure of the carbon black phase. XPS analysis indicated an increase in graphitic carbon at the electrode surface, consistent with the removal of surface polymer, which was further corroborated by SEM imaging. These surface modifications facilitate more efficient electric double‐layer formation, resulting in improved capacitance.

To understand further the effect of activation on the electrochemical performance of the CB/HDPE AMEP, cyclic voltammograms at 0.05 V s^−1^ before and after the activation process in [Ru(NH_3_)_6_]^3+^ and [Fe(CN)_6_]^4−^ were performed (Figure 4C,D). Testing both inner‐sphere and outer‐sphere redox probes provides crucial information on electrode behavior, whereby outer‐sphere probes assess the composite’s intrinsic conductivity, whereas inner‐sphere probes evaluate surface chemistry [42]. It can be observed that the activation process has significant effect on the HDPE electrodes (Figure 4C), where the peak current significantly increases post‐activation for [Ru(NH_3_)_6_]^3+^. This increase is interesting as it is not a characteristic usually seen with additive manufactured electrodes and outer‐sphere redox probes. This indicates the removal of significant amounts of surface polymer, confirmed by SEM, allowing for an increase in surface graphitic carbon, resulting in an increased current response. Polymers, such as PLA, do not show a significant increase toward outer‐sphere probes following activation, likely as PLA allows for large amount of ingress leading to a less dramatic increase [15, 22]. Polypropylene has shown that electrochemical activation minimally changes the surface structure of the electrode and therefore demonstrates no significant change electrochemically toward outer‐sphere probes post‐activation [28]. As expected, activation shows to be essential for CB/HDPE AMEP when used with an inner‐sphere probe such as [Fe(CN)_6_]^4−^, as it is evident that without activation, no redox peaks are observed (Figure 4D), suggesting that the conductive filler is fully covered by a layer of insulating polymer, hindering any interaction with the inner‐sphere probe. This agrees with the results obtained from SEM and XPS analysis, which showed a significant increase in graphitic carbon and decrease in surface polymer post‐activation.

To evaluate the heterogeneous electron charge transfer rate (*k* ^0^) and electrochemically active surface area (*A*_*e*_) of the activated CB/HDPE AMEP, a scan rate study was performed. Cyclic voltammetry was conducted over a range of scan rates (0.005–0.5 V s^−1^) using both an outer‐sphere probe ([Ru(NH_3_)_6_]^3+^) and an inner‐sphere probe ([Fe(CN)_6_]^4−^). The corresponding voltammograms are shown in Figure S7. For the CB/HDPE AMEP in 1 mM [Ru(NH_3_)_6_]^3+^ (0.1 M KCl), a *k* ^0^_obs_ of 2.93 (±0.19) × 10^−3^ cm s^–1^ was obtained. When benchmarked against other carbon black‐based additive manufactured electrodes fabricated in identical geometries, the CB/HDPE AMEP outperformed those reported in the literature. Reported values include 2.00 (±0.04) × 10^−3^ cm s^−1^ for CB/PP, 1.71 (±0.19) × 10^−3^ cm s^−1^ for CB/PLA, and 2.69 (±0.10) × 10^−3^ cm s^−1^ for CB/TPU [28, 30, 31]. In addition, the CB/HDPE AMEP exhibited an enhanced electrochemically active surface area of 0.50 (±0.01 cm^2^), exceeding that of CB/PP AMEP (0.45 cm^2^) reported under comparable conditions [28]. This increase in the electrochemically active surface area is consistent with the improved electrochemical performance observed and is likely due to the higher carbon exposure and more effective conductive pathways formed within the HDPE matrix. Consistent trends are evident for the inner‐sphere redox probe [Fe(CN)_6_]^4−^ (1 mM in 0.1 M KCl), where this exhibits a fast *k* ^0^_obs_ of 2.10 (±0.04) × 10^−4^ cm s^−1^ relative to values typically reported for additive manufactured electrodes, including those based on commercial conductive PLA. A comparatively high electrochemically active surface area of 0.47 (±0.06 cm^2^) was also obtained. Figure S7B shows well‐defined redox peaks for this system, with a peak‐to‐peak separation (Δ*E*_p_) of 275 mV at a scan rate of 0.025 V s^−1^, indicating good electrochemical responsiveness toward inner‐sphere species. However, when benchmarked against other carbon black–polymer composites, the CB/HDPE electrodes exhibit a larger Δ*E*_p_, suggesting comparatively slower electron transfer kinetics for this probe. Specifically, reported Δ*E*_p_ values for CB/PP and CB/PLA are 185 and 110 mV, respectively [13, 15].

Electrochemical characterization was further conducted using electrochemical impedance spectroscopy to evaluate the solution resistance (*R*_s_) and charge transfer resistance (*R*_CT_) of the CB/HDPE electrodes via equivalent circuit fitting. Measurements were performed in triplicate in a solution comprising 1 mM [Fe(CN)_6_]^/4−^ in 0.1 M KCl. The CB/HDPE AMEP exhibited favorable values for both parameters, with an *R*_s_ of 147 (±52) Ω, in good agreement with the filament resistances reported in Section 3.1. The *R*_CT_ was determined to be 14.4 (±2.5) kΩ, representing an improvement over CB/PP AMEP of 15.7 (±2.8) kΩ. This reduction in charge transfer resistance is likely attributable to enhanced exposure of carbon black at the electrode surface, facilitating more efficient interfacial electron transfer [43]. Following electrochemical characterization, it is evident that CB/HDPE represents a valuable addition to the electrochemist’s materials portfolio. The electrodes demonstrate improvements across several key performance metrics, most notably enhanced electron transfer kinetics. When coupled with the inherent mechanical robustness and chemical resistance of the HDPE matrix, these attributes underscore the suitability of CB/HDPE as a reliable feedstock for next‐generation additive manufactured electrochemical devices.

### 2.3 Stability Testing

To further assess the broader applicability of this material, the evaluation of long‐term stability under varying conditions is essential. Exposing the electrodes to different environments provides insight into their operational limits, durability, and suitability for repeated use prior to performance degradation or failure. As an initial stability assessment, CB/HDPE AMEP were subjected to 100 cyclic voltammetric scans at 25 mV s^−1^ in 1 mM [Ru(NH_3_)_6_]^3+^ (0.1 M KCl), corresponding to a cumulative duration of approximately 3 h. The resulting data are shown in Figure S8A. Notably, after 60 scans, the electrodes retained 90 (±2.66)% (*n* = 3) of their initial current response, demonstrating a high degree of electrochemical stability and reusability. Following 100 scans, a peak current reduction of 25.34 (±1.57)% (relative to the first scan) was observed, which represents a significant improvement compared to CB/PP AMEP, where a 52.4% decrease in response has been reported [43]. Figure S8B shows a comparison of the voltammograms at the 1st and 60^th^ scan, where it can also be seen that there is no change in peak potential, suggesting that the electrode maintains its electronic conductivity and integrity without significant degradation during repeated electrochemical cycling. Crucially, the potential to reliably reuse electrodes multiple times before failure can have a significant impact on the sustainability of products, synergising with the ultralow waste generation of additive manufacturing.

Stability in aqueous environments is critical, particularly for sensing applications; however, extending the scope of additive manufactured electrochemical systems requires robustness in more aggressive aqueous conditions as well as organic media. To this end, the chemical stability of CB/HDPE electrodes was systematically evaluated. Electrodes were immersed (in triplicate) for 70 days in six representative media: water, 0.5 M H_2_SO_4_, 1 M KOH, acetonitrile, dichloromethane (DCM), and N,N‐dimethylformamide (DMF). These solvents were selected based on their relevance to energy conversion and electrosynthetic applications. Mass measurements were recorded at regular intervals to assess solvent ingress and overall material stability. The results are shown in Table 1. Negligible changes in mass were observed over the 70‐day period in water, 0.5 M H_2_SO_4_, 1 M KOH, acetonitrile, and DMF, indicating excellent resistance to solvent ingress and strong chemical stability under both acidic, alkaline, and polar organic conditions. In contrast, exposure to DCM resulted in an initial mass increase of 4.84% within the first 24 h, followed by stabilization for the remainder of the time period. This behavior is consistent with solvent uptake and polymer swelling, likely arising from the similarity in solubility parameters between DCM and polyethylene, which promotes partial miscibility. Conversely, highly polar solvents, such as acetonitrile and DMF, exhibit minimal interaction with the non‐polar HDPE matrix, resulting in negligible swelling. These findings are significant, demonstrating that CB/HDPE electrodes can withstand chemically demanding environments without substantial degradation. Their resilience in strong acids, bases, and a range of organic solvents highlights their suitability for diverse electrochemical applications, including sensing platforms, energy storage and conversion systems, and electrosynthetic processes. Overall, the demonstrated chemical stability positions CB/HDPE as a versatile and robust alternative to other conductive polymer‐based electrode materials.

**TABLE 1:** The weight change in % for CB/HDPE AMEP across a 70‐day period in water, 0.5 M H_2_SO_4_, 1 M KOH, acetonitrile, DCM, and DMF.

Solvent	0.25 days	1 day	70 days
Water	0.07 ± 0.08	0.00 ± 0.01	0.01 ± 0.01
KOH (1 M)	0.46 ± 0.01	0.98 ± 0.01	0.29 ± 0.02
H_2_SO_4_ (0.5 M)	1.10 ± 0.01	1.11 ± 0.02	0.84 ± 0.02
MeCN	0.43 ± 0.03	0.11 ± 0.10	0.32 ± 0.01
DCM	4.54 ± 1.61	4.84 ± 1.18	5.28 ± 1.76
DMF	1.55 ± 0.71	3.08 ± 1.52	3.05 ± 1.80

*Note:* Data are presented as mean ± standard deviation (*n* = 3).

Abbreviation: DCM, dichloromethane; DMF, dimethylformamide; MeCN, acetonitrile.

## 3 Development of Electrochemical Applications

Having demonstrated the electrochemical performance and chemical resilience of the CB/HDPE filament and associated AMEPs, we demonstrate their versatility through two proof‐of‐concept applications: the electrosynthetic transformation of chlorpromazine to its sulfoxide in acetonitrile and the electroanalytical determination of caffeine in commercial beverages. These applications demonstrate the potential of CB/HDPE AMEP to support both synthetic and analytical electrochemistry.

### 3.1 Electrosynthesis of Chlorpromazine in Organic Solvent

To highlight the performance of the CB/HDPE within non‐aqueous electrolytes, it was applied toward the electrosynthetic transformation of CPZ to CPZ sulfoxide within acetonitrile. CPZ is a first‐generation, low‐potency typical antipsychotic agent to manage specific mental disorders. The antipsychotic properties of CPZ are primarily achieved through the blockade of dopaminergic receptors D_1_ to D_4_, with a particular focus on the D_2_ receptor. The use of CPZ is associated with several notable adverse effects, including extrapyramidal symptoms, orthostatic hypotension, sedation, and ocular complications [44]. Clinical studies indicate that bioactive metabolites of CPZ contribute significantly to toxicity in humans, leading to these side effects [44]. The primary metabolites of CPZ in humans include 7‐hydroxy‐CPZ, N‐monodesmethyl‐CPZ, and CPZ sulfoxide [45]. The existence of these circulating metabolites affects the therapeutic properties of pharmaceuticals, and in turn, methods to efficiently synthesize drug metabolites for biological evaluation are in demand [46]. The electrochemical behavior and electrosynthesis of CPZ metabolites have been previously studied using CB/PP additive manufactured electrodes, which showed that CPZ sulfoxide is the major metabolite generated using both glassy carbon and CB/PP electrodes [47]. CB/PP performed similarly to the GCE, achieving a near threefold improvement in the ratio between CPZ sulfoxide and CPZ sulfone (11:1 to 27:1 ratio) [28]. Given the enhanced chemical stability and electrochemical performance of the CB/HDPE, the same electrosynthetic methodology was applied to assess the feasibility of using CB/HDPE AMEPs for CPZ oxidation and to evaluate the resulting product distribution relative to previously reported 30 wt% CB/PP AMEPs [13] (Scheme 1 and Table 2).

**SCHEME 1:**
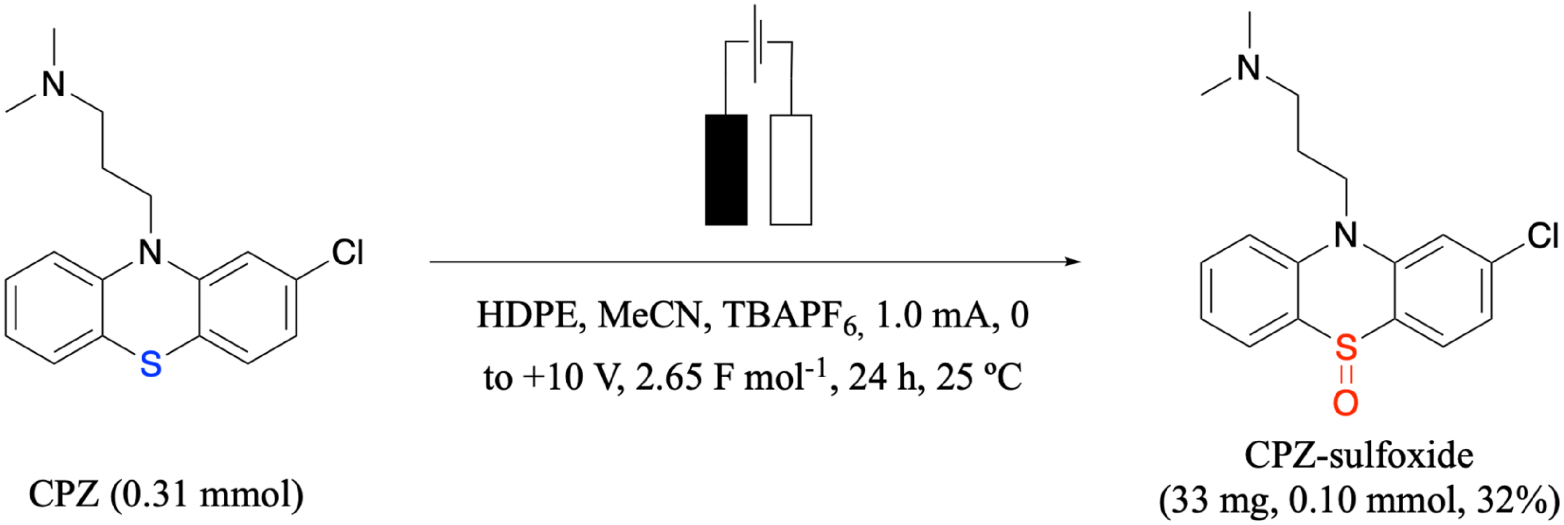
Schematic of CPZ sulfoxide electrosynthesis reaction using ElectraSyn 2.0 (IKA). CPZ (0.31 mmol), electrode: (+) additively manufactured CB/HDPE electrodes (−) additively manufactured CB/HDPE electrodes. Electrolyte: TBAPF_6_, solvent: MeCN, 1 mA, 24 h, 25 ºC.

**TABLE 2:** The comparison between the commercial GCE electrode and the custom‐made electrode CB/PP, and CB/HDPE in the electrosynthesis of CPZ metabolites (CPZ sulfoxide (CPZSO) and CPZ sulfone (CPZSO_2_).

Entry	Electrodes	**Max voltage applied,** **V**	**Total charged,** **F mol** ^**−1**^	Metabolite ratio
				**CPZ/CPZSO/CPZSO**_**2**_
1	GCE	0 to +3.86	2.81	1:335:30
2	30% CB/PP	0 to +7.33	2.62	1:82:3
3	aCB/HDPE	0 to +3.48	2.95	1:60:9
4	bCB/HDPE	0 to +10.00	2.65	1:155:21

*Note:* Pretreatment conditions: Additively manufactured CB/HDPE electrodes were treated with 0.5 M NaOH solution using chronoamperometry set to +1.4 V (vs. Ag/AgCl) for 200 s, followed by −1.0 V (vs. Ag/AgCl) for 200 s.

a HDPE without pretreatment.

b HDPE with pretreatment.

The CB/HDPE AMEP successfully supported CPZ oxidation during 24 h of galvanostatic electrolysis in acetonitrile, with no visible electrode degradation and an isolated CPZ sulfoxide yield of 32% (supporting data are shown in Figures S9–S17). Activation increased the CPZ sulfoxide‐to‐residual CPZ ratio from 60:1 to 155:1, although the total charge passed was comparable and the CPZ sulfoxide‐to‐CPZ sulfone selectivity increased only slightly, from 6.7:1 to 7.4:1. The activated CB/HDPE cell also required a comparatively high maximum cell voltage of 10.0 V, indicating an energetic limitation associated with cell resistance and polarization. Although CB/HDPE was more brittle than CB/PP, these results demonstrate its feasibility and chemical stability as an electrode material for prolonged non‐aqueous electrosynthesis.

### 3.2 Electroanalytical Detection of Caffeine in Aqueous Solution

CB/HDPE AMEP was employed for the electroanalytical detection of caffeine, a target of significant relevance given that approximately 80% of the global population consumes caffeine daily, making it the most widely used stimulant [48]. Although moderate consumption is generally considered safe, excessive intake can lead to adverse health effects. The European Food Safety Authority (EFSA) recommends a maximum daily intake of 400 mg for healthy adults, with lower limits advised for adolescents and pregnant women [49]. Notably, studies have indicated that caffeine consumption exceeding 200 mg per day during pregnancy may increase the risk of miscarriage [50]. This establishes the significance for the development of reliable sensing technology for caffeine analysis, especially at point of use, which can provide with rapid and low‐cost alternatives to traditional analytical methods. Furthermore, caffeine is a well‐established model analyte in electrochemical research, enabling direct benchmarking of CB/HDPE electrode performance against existing systems and providing a benchmark for their electrochemical sensing abilities. A study was performed on the pH on the electrochemical oxidation of caffeine in terms of peak current and peak potential across the pH range for 2–9, where we observed that pH gave the largest response and it can be seen that the peak did not shift with pH. Caffeine remains essentially neutral due to its pK_a_ ~0.7, meaning that it remains in its neutral form and will not see a Nerstian pH dependence shift [51]. The electrochemical oxidation of caffeine has been reported to proceed via an initial one‐electron transfer, forming a radical cation, which subsequently undergoes nucleophilic attack by water or buffer species [52]. The absence of peak potential shift with pH suggests that proton transfer is not involved in the rate‐determining electrochemical step, consistent with a radical cation formation mechanism.

Differential pulse voltammetry was selected for caffeine detection due to its enhanced sensitivity and reduced capacitive background current relative to cyclic voltammetry. The standard addition method was employed to construct a calibration curve over the concentration range from 10 to 300 μM, Figure 5, yielding a linear response described by: I (μA) = 0.18_[CAF]_ + 1.23, *R* ^2^ = 0.998. From this calibration, the limit of detection (LOD) and limit of quantification (LOQ) were determined to assess the lowest reliably measurable analyte concentrations using the additive manufactured electrodes. The CB/HDPE AMEP exhibited an LOD of 0.14 μM and an LOQ of 0.47 μM. While the CB/HDPE AMEP exhibited excellent performance for caffeine sensing under laboratory conditions, it is essential to evaluate their applicability in real samples. To this end, the electrodes were employed to detect caffeine in commercial beverages of Red Bull energy drink sugar‐free and Starbucks doubleshot espresso. The caffeine concentration in each sample was first validated using liquid chromatography mass spectroscopy (LC‐MS) to obtain accurate quantitative values before applying the electrochemical method; further details are summarized in the ESI where one can see the LC‐MS parameters, Tables S2 and S3. As shown in Table S4, we compare LC‐MS against our electrochemical approach which are applied to the Red Bull sample (diluted 20:1), the electrodes achieved a 98 (±2)% recovery, while a 102 (±3)% recovery was obtained for the espresso sample (diluted 50:1). Figure S18 shows the voltammograms and calibration plots for both samples, which both showed to be linear. The Red Bull sample resulted in a linear response corresponding to I (μA) = 0.08_[CAF]_ + 5.80, *R* ^2^ = 0.999, and the espresso sample provided a linear response of I (μA) = 0.01_[CAF]_ + 0.32, *R* ^2^ = 0.974. The excellent recovery values confirm the suitability of these electrodes for real‐sample analysis, and the overall performance highlights the potential of the CB/HDPE AMEP for applications within this field.

**FIGURE 5:**
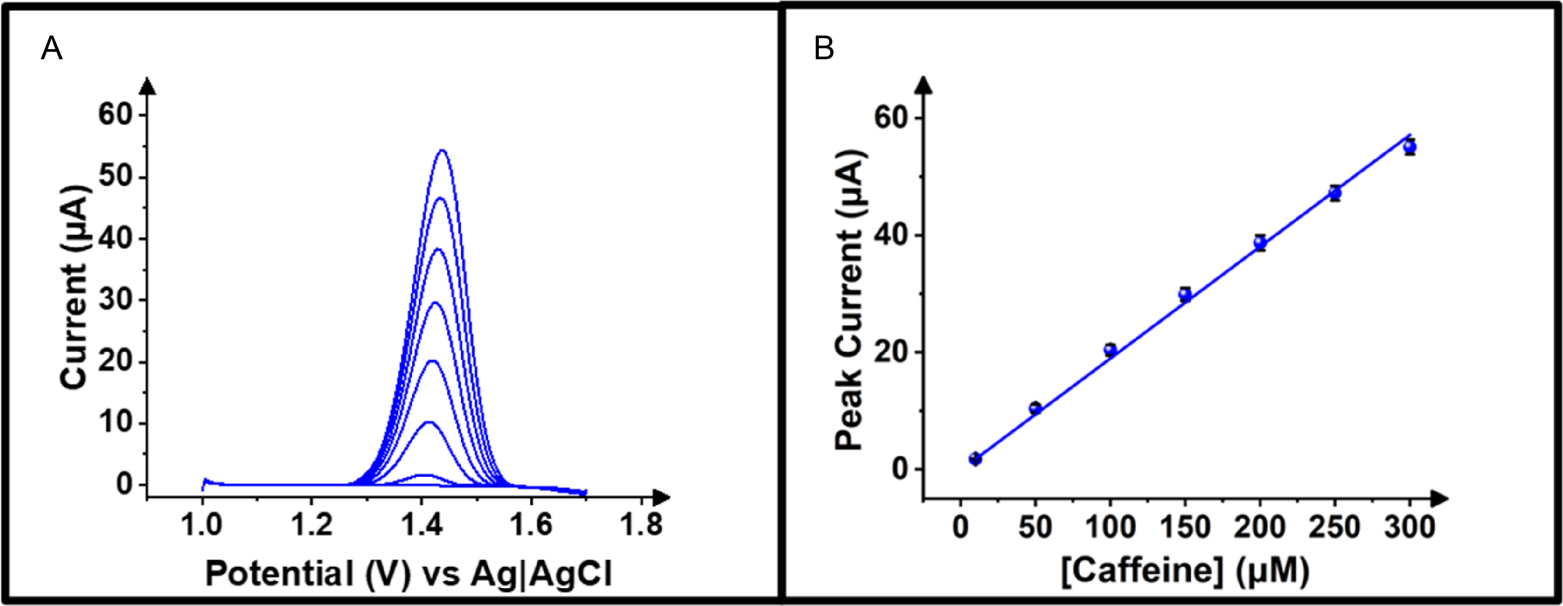
(A) Differential pulse voltammogram for the CB/HDPE AMEP toward the detection of caffeine in a concentration range from 10 to 300 μM in a pH5 BR buffer. (B) Calibration plot for peaks obtained for caffeine when using CB/HDPE AMEP. Conditions applied for DPV are as follows: potential range = 0–1.7 V, step potential = 0.005 V, modulation amplitude = 0.05 V, modulation time = 0.05 s, interval time = 0.5 s. (*N* = 3).

## 4 Conclusion

In this work, we demonstrate, for the first time, a conductive HDPE filament for engineering AMEP. Importantly, the as‐printed electrode must be distinguished from the functional electrochemical platform: Post‐print electrochemical activation is required to expose the conductive carbon black phase. This step is particularly important for inner‐sphere redox processes, for which the polymer‐rich surface of the as‐printed electrode otherwise inhibits electron transfer. The filament contains no plasticizers but remains sufficiently flexible and printable without requiring their addition. The novelty of the study therefore lies in establishing HDPE as a chemically resistant conductive matrix for AMEP rather than in proposing a new FFF process or electrode geometry.

The activated electrodes demonstrated good operational stability during repeated cyclic voltammetry. After 60 and 100 scans, the peak current response decreased by 10.0 (±2.66)% and 25.34 (±1.57)%, respectively, relative to the initial scan. Long‐term chemical stability was also evaluated by immersing the electrodes for 70 days in water, 0.5 M H_2_SO_4_, 1 M KOH, acetonitrile, dichloromethane, and N,N‐dimethylformamide. Gravimetric analysis revealed minimal changes in mass in most of the tested environments, demonstrating good chemical stability under the conditions investigated. The activated CB/HDPE platforms were successfully applied to both electrosynthetic and electroanalytical applications. This work provides a new polymer for additive manufactured electrochemical devices, enabling operation in both aqueous and non‐aqueous environments and expanding the scope of applications previously inaccessible to conventional polymeric‐based materials.

## Conflicts of Interest

The authors declare no conflicts of interest.

## Supporting information

Supplementary Material

Supplementary Material

## Data Availability

The data that support the findings of this study are available from the corresponding author upon reasonable request.
