# Supplementary material for: Additive Manufacturing Engineering for Electrochemistry: Conductive High‐Density Polyethylene Filament Expands the Materials Landscape
Source: Small Sci. 2026 Oct 8;6(10):e70425. doi: 10.1002/smsc.70425 (PMC13650387; doi:10.1002/smsc.70425)
Supplement: Supplementary file 1 [file SMSC-6-e70425-s002.pdf]

Supplementary Information for:

Additive Manufacturing Engineering for Electrochemistry: Conductive High-Density Polyethylene Filament Expands the Materials Landscape

Muhzamil A. Khan,[1] Elena Bernalte,[1] Ridho Asra,[2] Robert S. Tapping,[1] Ana C.M. Oliveira,[1,3] Alan M. Jones,[2] Rodrigo A. A. Muñoz,[3] Samuel Rowley-Neale,[1] Robert D. Crapnell,[1*] and Craig E. Banks[1*]

[1] *Faculty of Science and Engineering, Manchester Metropolitan University, Dalton Building, Chester Street, M1 5GD, Great Britain.*

[2] *School of Pharmacy, School of Health Sciences, College of Medicine and Health, University of Birmingham, Edgbaston, B15 2TT, Great Britain*

[3] *Institute of Chemistry. Federal University of Uberlândia, Uberlândia, Minas Gerais 38400-902, Brazil*

[*]To whom correspondence should be addressed.

E-mail: r.crapnell@mmu.ac.uk; c.banks@mmu.ac.uk

Tel: +44(0)1612471196

Experimental Section

Chemicals

All chemicals were of analytical grade and were used as received without any further purification. All solutions were prepared with deionised water of resistivity not less than 18.2 MΩ cm from a Milli-Q Integral 3 system from Millipore UK. Hexaamineruthenium (III) chloride (98%), potassium ferricyanide (99%), potassium ferrocyanide (98.5%), potassium chloride (99%), sodium hydroxide (97%), acetonitrile (99.8%), dichloromethane, N,N-dimethylformamide (99.8%), sulfuric acid (99.9%), acetic acid (99.9%), boric acid (99.5%), phosphoric acid (99.9%) and potassium hydroxide (99.9%) were purchased from Sigma (Gillingham, UK). Caffeine (98.5%) was purchased from Alfa Aesar (Loughborough, UK). Carbon black (CB) was purchased from PI-KEM (<20 nm, >99+%, Tamworth, UK), High density poly(ethylene) (HDPE) pellets were purchased from Hardie Polymers (Hostalen GC 7260 HDPE; Melt Flow Index of 8.0g/10min, Milngavie, Glasgow, UK). Poly(ethylene) tape was purchased from Banner (Wakefield, UK).

Bespoke Filament Production

Prior to the filament production, all HDPE pellets were dried in an oven at 80 °C for a minimum of 2.5 h to remove any residual water inside the polymer. The bespoke conductive filament was prepared using 60 wt% HDPE and 40 wt% CB without the need of additional plasticiser. The compounds were mixed at 210 °C with Banbury rotors for 5 min using a Thermo Haake Polydrive dynameter fitted with a Thermo Haake Rheomix 600 (Thermo-Haake, Germany). The resulting polymer composite was allowed to cool to room temperature before being granulated using a Rapid Granulator 1528 (Rapid, Sweden). Next, the samples were collected and processed through the hopper of a EX2 extrusion line (Filabot, VA, USA) set to 210 °C. The molten polymer was extruded from a 1.75 mm die head, pulled along an Airpath cooling line (Filabot, VA, USA). The filament was ready for additive manufacturing.

Additive Manufacturing of Electrodes

All computer designs and .3MF files in this manuscript were produced using Fusion 360® (Autodesk®, CA, USA). These files were sliced and converted to .GCODE files for printing by the open-source software, PrusaSlicer (Prusa Research, Prague, Czech Republic). The additively manufactured electrodes were 3D-printed using fused filament fabrication (FFF) technology on a Prusa i3 MK3S+ (Prusa Research, Prague, Czech Republic). All additively

manufactured electrodes were printed onto poly(ethylene) tape using a 0.6 mm nozzle with a nozzle temperature of 210 °C and bed temperature of 100 °C, 100% rectilinear infill [1], 0.15 mm layer height, and print speed of 35 mm s^{-1}.

Physicochemical Characterisation

Thermogravimetric analysis (TGA) was performed using a Discovery Series SDT 650 instrument controlled by Trios Software (TA Instruments, DE, USA). Samples were mounted in alumina pans (90 μL) and tested using a ramp profile (10 °C min^{-1}) from ambient conditions to 800 °C under N_2 (100 mL min^{-1}). In separate experiments, with the same instrument, differential scanning calorimetry (DSC) measurements were taken for the raw HDPE, non-conductive HDPE/Electrode and CB/HDPE/Electrode samples from ambient conditions to 170 °C at a rate of 2.5 °C min^{-1} under a 100 mL min^{-1} N_2 flow. The heat of fusion (ΔH_m) was calculated by first determining the onset temperature of the melting transition and integrating the peak using a spline like baseline. From this the crystallinity of the HDPE can be determined using $X_c = (\Delta H_m / (1 - w) \Delta H^0_m)\ 100$, where w is the weight fraction of CB, i.e. 0.4 or 40% and ΔH^0_m is the heat of fusion of 100% crystalline HDPE (293 J g^{-1}) [2].

X-ray diffraction (XRD) patterns were collected on a PANalytical X'pert Powder X-ray diffractometer using Cu Kα1 radiation (λ = 1.5406 Å) using a PIXCel 1-D detector. The samples were rotated at 16 rpm during the data collection. A selected HDPE bead was chosen that resembled a similar shape to the electrodes and contained a flat edge. After analysis, unit cell parameters were found using Profex V5.5 refinement software. The background was modelled by the implemented improved asymmetrically reweighted penalized least squares function on GSAS II with a Log Lambda value of 8.0. Since the atomic positions of carbon and hydrogen in HDPE are well known [3], a full structure refinement was deemed not necessary, therefore LeBail fits were performed on each sample in the range of 5-140 2θ to extract unit cell parameters and coherence lengths. A test for intensity correction using spherical harmonics was used from orders 0-10. The result in all cases suggested a decrease in R_{wp} and G_{of} from order 0-2; however, from 2-10 the decreases were marginal. Therefore, a spherical harmonics order of 2 was used which also aligns with the axial symmetry and chain-like structure [3]. To model the HDPE phase accurately, either one or two amorphous phases were added at either 20 or 25.5 2θ that represent the amorphous HDPE and carbon black, respectively [4]. Coherence lengths were determined using the implemented GrainSize(h,k,l) function implemented into Profex V6. All atomic models were made on VESTA.

Scanning electron microscopy (SEM) micrographs were obtained using a Crossbeam 350 Focussed Ion Beam–scanning electron microscope (FIB-SEM) (Carl Zeiss Ltd., Cambridge, UK) fitted with a field emission electron gun. Imaging was completed using a secondary electron secondary ion (SESI) detector. Samples were mounted on the aluminium SEM pin stubs (12 mm diameter, Agar Scientific, Essex, UK) using adhesive carbon tabs (12 mm diameter, Agar Scientific, Essex, UK) and coated with a 3 nm layer of Au/Pd metal using a Leica EM ACE200 coating system before imaging.

Raman spectroscopy was performed on a Renishaw PLC in Via Raman Microscope controlled by WiRE 2 software at a laser wavelength of 514 nm.

X-ray photoelectron spectroscopy (XPS) data were acquired using an AXIS Supra (Kratos, UK), equipped with a monochromated Al X-ray source (1486.6 eV) operating at 225 W and a hemispherical sector analyser. It was operated in fixed transmission mode with a pass energy of 160 eV for survey scans and 20 eV for region scans with the collimator operating in slot mode for an analysis area of approximately 700 × 300 µm, the FWHM of the Ag 3d5/2 peak using a pass energy of 20 eV was 0.613 eV. Before analysis, each sample was ultrasonicated for 15 min in propan-2-ol and dried for 2.5 h at 65 °C as shown in our unpublished data to remove excess contamination and minimise the risk of misleading data. The binding energy scale was calibrated by setting the graphitic sp^2 C 1s peak to 284.5 eV; this calibration is acknowledged to be flawed but was nonetheless used in the absence of reasonable alternatives, and because only limited information was to be inferred from absolute peak positions [5].

Contact angles were assessed using a bespoke additively manufactured measurement set-up consisting of an additive manufactured stage, a Hamilton syringe, and a generic USB digital microscope connected to a computer. This equipment allowed consistent delivery of droplets of deionised water to part surfaces which could be recorded as video and/or photographs using the microscope. Where possible, contact angles were analysed using ImageJ and the DropSnake plug-in, with reported values being the average and standard deviation of three repeat measurements.

Electrochemical Characterisation

Electrochemical measurements were performed on an Autolab 100N and 128N potentiostat controlled by NOVA 2.1.8 (Utrecht, the Netherlands). The electrochemical characterisation of the bespoke filament and comparison to the benchmarks were performed using a lollipop design (Ø 5 mm disc with 8 mm connection length and 2 × 1 mm thickness) electrodes alongside an external commercial Ag|AgCl (3 M KCl) reference electrode with a nichrome wire counter electrode.

Electrochemical activation of the additively manufactured electrodes was performed before the electrochemical experiments. The additively manufactured electrodes were used as a working electrode alongside a nichrome wire coil as a counter electrode and an Ag|AgCl (3 M KCl) as a reference electrode and placed into a 0.5 M NaOH solution. Chronoamperometry was used to activate the additively manufactured electrodes by applying a set voltage of +1.4 V for 200 s, followed by applying −1.0 V for 200 s. The additively manufactured electrodes were after thoroughly rinsed with deionised water and dried under compressed air before further use.

Cyclic voltammetry was applied to carry out a scan rate study on the additively manufactured electrodes varying scan rates from 0.005 - 0.5 V s^{-1} were applied in both an outer-sphere redox probe of $[Ru(NH_3)_6]^{3+}$ (1 mM in 0.1 M KCl) and inner-sphere probe of $[Fe(CN)_6]^{4-}$ (1mM in 0.1 M KCl). All solutions of $[Ru(NH_3)_6]^{3+}$ were thoroughly purged of O_2 prior to electrochemical analysis with N_2. Interpretation of the data was completed through use of the Nicholson equation (Equation 1) which allowed for the calculation of the heterogenous electron transfer rate (k^0_{obs}). The parameters applied to the Nicholson equation are as listed:

$$\Psi = k^0_{obs} (\pi DnvF/RT)^{-1/2} \quad (1)$$

where Ψ is a kinetic parameter, D is the diffusion coefficient ($[Ru(NH_3)_6]^{3+}$ = 9.10 x 10^{-6} cm^2 s^{-1}, $[Fe(CN)_6]^{4-}$ = 6.50 x 10^{-6} cm^2 s^{-1}), n is the number of electrons that are taking part in the redox reaction, F is the Faraday constant (96485 C mol^{-1}), v is the scan rate (5-500 mV s^{-1}), R is the universal gas constant (8.314 J mol^{-1} K^{-1}) and T is the temperature in Kelvin (298 K). In order to calculate k^0_{obs}, determination of the peak-to-peak separation (ΔE_p) must be completed to deduce Ψ, where ΔE_p is obtained at various voltametric scan rates. The k^0_{obs} can be obtained via the gradient when plotting Ψ against $[\pi DnvF/RT]^{-1/2}$. The electrochemically active surface area (A_e) was determined from the appropriate Randles-Ševčík equation.

Capacitance of the electrodes was calculated through use of cyclic voltammetry in the non-faradaic area. Electrodes were used in the standard electrochemical setup in 0.1 M KCl. The parameters used for cyclic voltammetry are as follows: start potential = +0.0 V; upper potential = +0.3 V; step potential = 0.0024 V; scan rate = 0.01-0.3 V s^{-1}.

Electrochemical impedance spectroscopy (EIS) was carried out in the standard electrochemical setup in $[Fe(CN)_6]^{3-/4-}$ (1mM in 0.1 M KCl) and recorded within a frequency range between 0.1 Hz to 100 kHz by applying 10 mV of signal amplitude to perturb the system under quiescent conditions. NOVA 2.1.7 software was used to fit Nyquist plots obtained to adequate equivalent circuit.

Aqueous Electroanalytical Application: Real samples' preparation

Real life samples were prepared differently according to the sample types. Prior to analysis the energy drink sample (Redbull®) was degassed for 15 minutes using a Grant Ultrasonic bath XUBA3 before being filtered through a 0.22 Millipore mixed cellulose ester syringe filter and diluted in a 1:20 ratio in a pH 5 Britton-Robinson (BR) buffer. Preparation of the coffee beverage sample (Starbucks doubleshot expresso®) was carried out by initially cooling the sample in an ice bath for 30 minutes to promote the solidification of lipids due to the beverage being a milk-coffee mix. The cooled sample is transferred to centrifuge tubes and centrifuged at 1800 xg for 15 minutes at 7°C using a Sigma 3-16KL refrigerated centrifuge. Following centrifugation, the supernatant phase were carefully collected and filtered through a 0.22 Millipore mixed cellulose ester syringe filter and diluted in a 1:50 ratio in a pH 5 BR buffer.

Validation using Liquid Chromatography – Mass Spectrometry (LC-MS)

Quantification of the caffeine content in the selected drink samples was completed using LC-MS. The instrument was an Agilent High-Pressure LC-Ultraviolet (UV)-MS Single Quadrupole (SQ) (HPLC-UV-MS) (1260 InfinityII). The column was an Agilent Eclipse Plus C18 2.1 x 50mm. Ionisation was completed using an electron-spray ionisation (ESI) method. Details of the acquisition method are provided in Table S2. The instrument was run in gradient flow mode using the timetable given in Table S3 Data was analysed using MassHunter software.

Organic Electrosynthetic Application

Chemicals and Methods

Solvents used for workup procedures were of HPLC grade from Sigma-Aldrich, VWR or Fisher Scientific. Unless stated otherwise, solvents were removed by rotary evaporation under reduced pressure between 30-50 °C. All chemical reagents were used as received unless stated otherwise. Reactions were monitored by TLC analysis on Merck silica gel 60 F254 using UV light (254 nm) and/or potassium permanganate. 1H and 13C NMR spectra were recorded either on a Bruker AVIII operating at 300 MHz for 1H and fitted with a 5mm BBFO probe or on a Bruker AVANCE NEO operating at 400 MHz for 1H fitted with a 5 mm "smart" BBFO probe, respectively. Chemical shift data for 1H are reported in parts per million (ppm, δ scale) downfield from tetramethylsilane (TMS: δ 0.0) and referenced internally to the residual proton in the solvent. The deuterated solvents used for NMR analysis were chloroform ($CDCl_3$:δH 7.26, δC 77.2), methanol (MeOD: δH 3.31, δC 49.2) and dimethyl sulfoxide (DMSO-d_6: δH 2.50, δC 39.5). Coupling constants (J) are given in hertz (Hz). The data are presented as follows: chemical shift, multiplicity (s = singlet, d = doublet, t = triplet, q = quartet, p = pentet, m = multiple, br = broad, app = apparent and combinations thereof), coupling constant and integration and assignment. Mass spectra were recorded on a Waters Xevo G2-XS ToF or Synap G2-S mass spectrometer using Zspray, Electro-spray ionization in negative (ESI-) and positive (ESI+) mode, respectively.

Sample preparation

Chlorpromazine (CPZ) (100 mg, 0.31 mmol) (Sigma Aldrich®) and tetrabutylammonium hexafluorophosphate ($TBAPF_6$) (608 mg, 1.57 mmol) (Sigma Aldrich®) (analyte: electrolyte 1:5) were dissolved in acetonitrile (MeCN) (12.0 mL) (Sigma Aldrich®).

Electrosynthesis of CPZ metabolites

Electrosynthesis of CPZ metabolites were performed using ElectraSyn 2.0 (IKA®) under a constant current control. An undivided glass cell (electrochemical vial) equipped with a magnetic stirrer was added to the analyte solution under study. Rectangular glassy carbon electrodes (GCE) (IKA®, Dimensions (W x H x D = 8 x 52.5 x 2 mm) as the control standard electrodes or same shape HDPE electrodes (Dimensions (W x H x D = 8 x 52.5 x 2 mm) as both the working electrode (WE) and the counter electrode (CE) were inserted into the solution

at an opposing distance of ~ 5 mm. Prior to the experiment, the electrodes were rinsed with double distilled, deionised water, followed by MeCN, and allowed to air-dry prior to the experiment. A fixed current (1.0 mA) was passed through the solution for 24 h with a stirring speed of 500 rpm. The electrolysis product was analysed and monitored using TLC (SiO_2, eluent – DCM: Methanol– 9:1) and the $TBAPF_6$ was removed by recrystallisation before HPLC analysis.

Recrystallisation of $TBAPF_6$

The reaction mixture in MeCN was decanted into the flask, and MeCN was evaporated using a rotary evaporator. Methanol (15 mL) was added to dissolve the crude and evaporated until crystals of $TBAPF_6$ formed. The crystals of $TBAPF_6$ were separated from the filtrate, and the filtrate was cooled overnight in the fridge (0 °C), and a secondary batch of $TBAPF_6$ crystals formed. The crystals of $TBAPF_6$ were collected either by filtration or using a Pasteur pipette to collect and separate the filtrate containing a mixture product of CPZ metabolites.

Metabolite purification

The purification of CPZ metabolites was performed using the Biotage® Isolera™ system as follows: Column: Biotage® Sfär silica high-capacity duo columns 20 µm (25 g) with Samplet®. Mobile phase: [A] DCM; [B] Methanol. Gradient: 0% B for 5 CV length to 10% B for 10 CV length; held for 15 CV. Flow rate: 35 mL min^{-1}. UV detectors: 254 and 280 nm.

HPLC Method and Results

The HPLC analysis conditions for CPZ metabolites are as follows: Column-Ascentis® C18 HPLC Column RP-Amide, 25 cm x 4.6 mm I.D., 5 µm particles (581325-U). Mobile phase: [A] 0.05% TFA in water; [B] acetonitrile. Gradient: 10 to 85% B in 16 min, held for 2 min, to 10% B in 1 min, held for 2.5 min. Flow rate: 1.0 mL min^{-1}. Injection: 5.0 µL. Chromatograms were recorded at 254 with a run time of 25 min.

Table S1. Atomic concentration (%) values of C 1s peak for the as printed and activated HDPE electrodes.

Sample	Graphitic	C-C	C-O	C=O	O-C=O
**As-printed**	1.05 (± 0.75)	83.89 (± 2.01)	9.54 (± 1.59)	2.50 (± 0.68)	3.01 (± 0.78)
**Activated**	36.07 (± 12.52)	57.54 (± 14.92)	4.50 (± 3.43)	0.89 (± 0.28)	0.99 (± 0.06)

Table S2. Instrumental parameters for the LC-MS.

Parameter	Value
**Run time**	25 minutes
**Draw speed**	200 μL min^{-1}
**Eject speed**	400 μL min^{-1}
**Wait time after drawing**	1.2 s
**Injection volume**	3.00 μL
**Solvent A**	0.01% Formic acid in water
**Solvent B**	Acetonitrile (HPLC grade)
**Column temperature**	25 ^{o}C

Table S3. Gradient timetable.

Time (min)	A (%)	B (%)	Flow (mL/min)	Max. pressure limit (Bar)
**0.00**	95	5	0.200	400
**3.00**	95	5	0.200	800
**5.00**	35	65	0.200	800
**9.00**	35	65	0.200	800
**9.10**	15	85	0.200	800
**12.00**	15	85	0.200	800
**12.10**	5	95	0.200	800
**15.00**	5	95	0.200	800
**15.10**	0	100	0.200	800
**20.00**	0	100	0.200	800
**20.10**	95	5	0.200	800
**25.00**	95	5	0.200	800

Table S4. Highlighting concentration of caffeine in Redbull and Espresso calculated via LC-MS and electrochemically. (n = 3).

Sample	LC-MS Calculated concentration (µM)	Dilution Factor	Electrochemical calculated concentration (µM)	Recovery (%)
**Red Bull**	1398.09 (± 1.49)	20:1	69.09 (± 0.69)	98 (± 2)
**Espresso**	1966.80 (± 2.72)	50:1	40.24 (± 1.63)	102 (± 3)

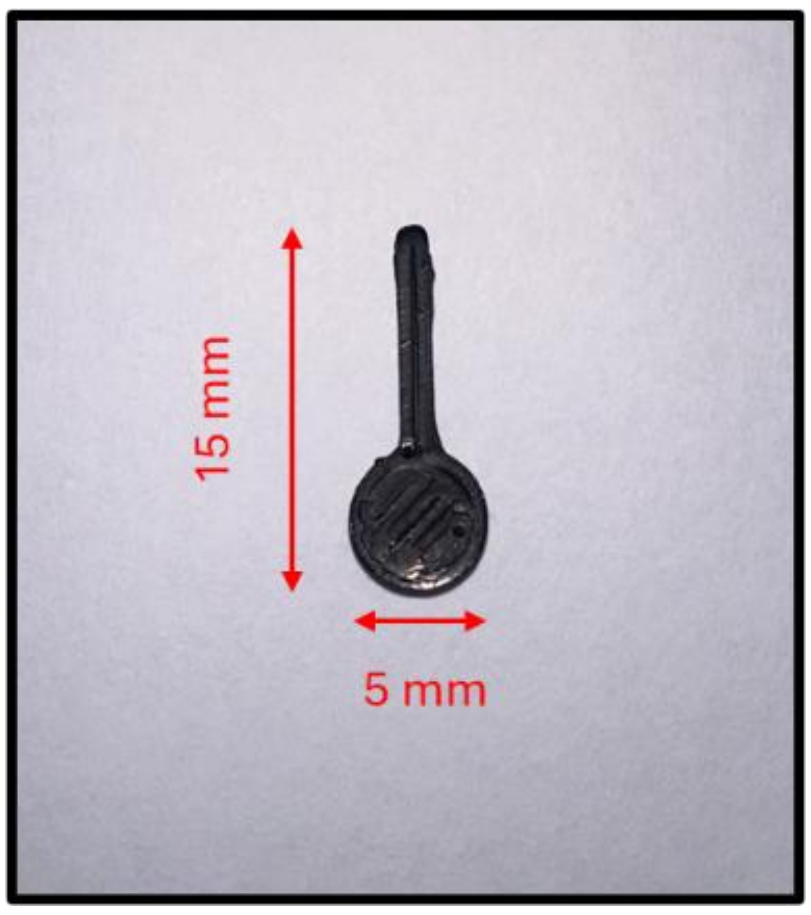

Figure S1. Image of the additively manufactured CB/HDPE electrode and its real dimensions. It has a 5 mm disc with 8 mm connection length and 2 × 1 mm thickness.

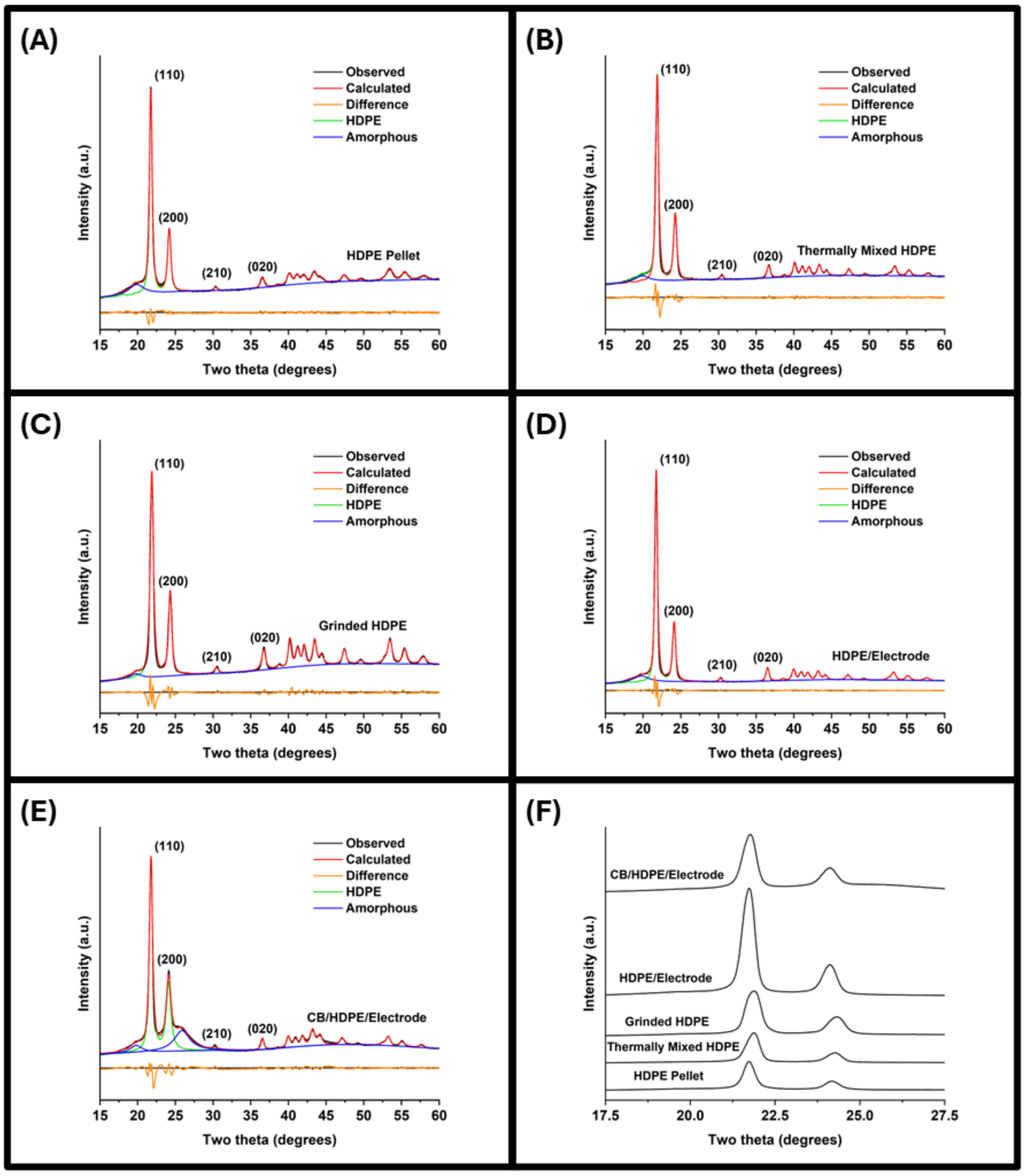

Figure S2. Whole powder pattern fitting of **A)** HDPE pellet, **B)** Thermally mixed HDPE, **C)** Grinded HDPE, **D)** additive manufactured HDPE electrochemical platform, **E)** additive manufactured CB/HDPE electrochemical platform and **F)** comparison of XRD patterns taken throughout the filament/electrode production process.

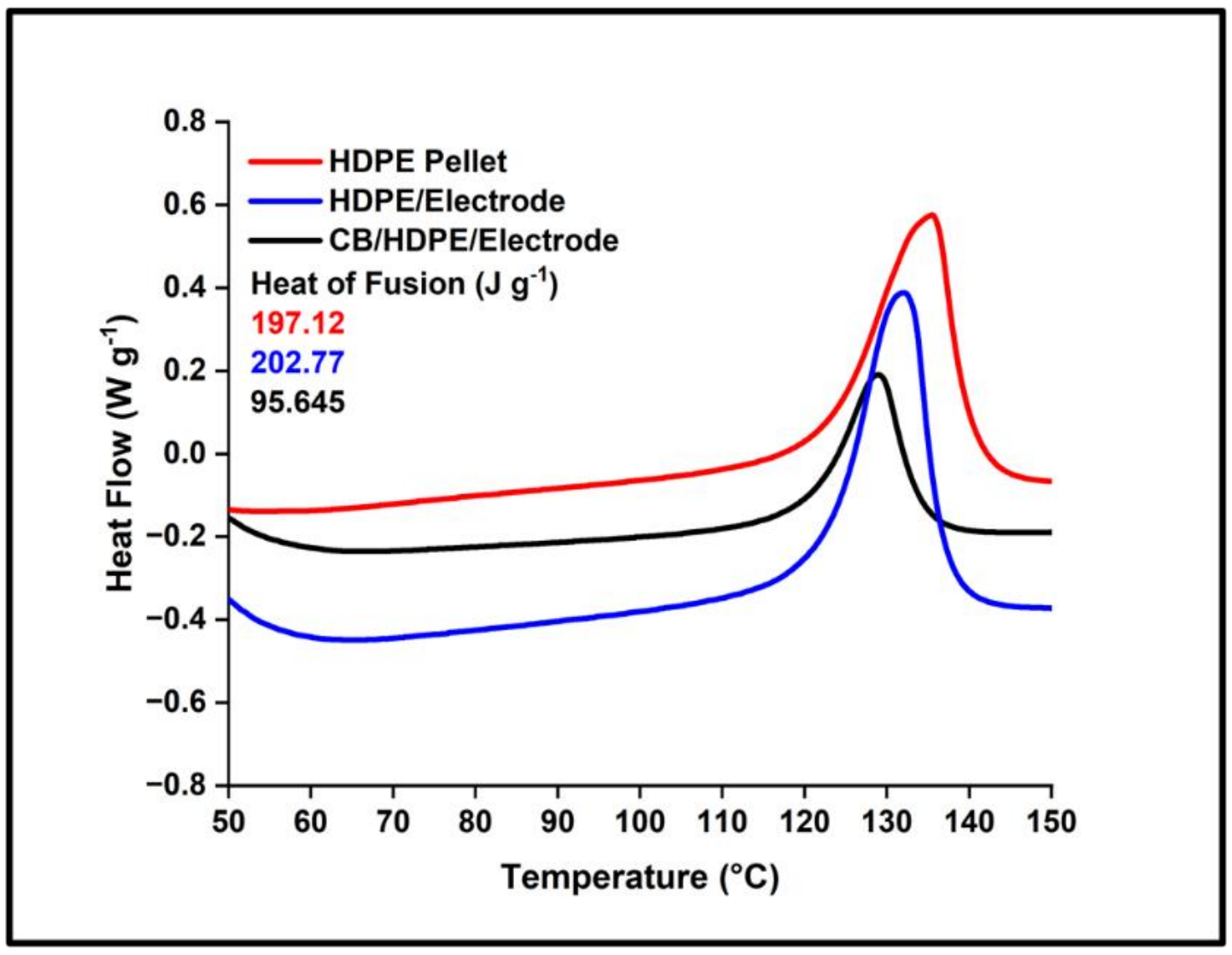

Figure S3. DSC measurements of HDPE pellet, additive manufactured HDPE electrochemical platform and additive manufactured CB/ HDPE electrochemical platform.

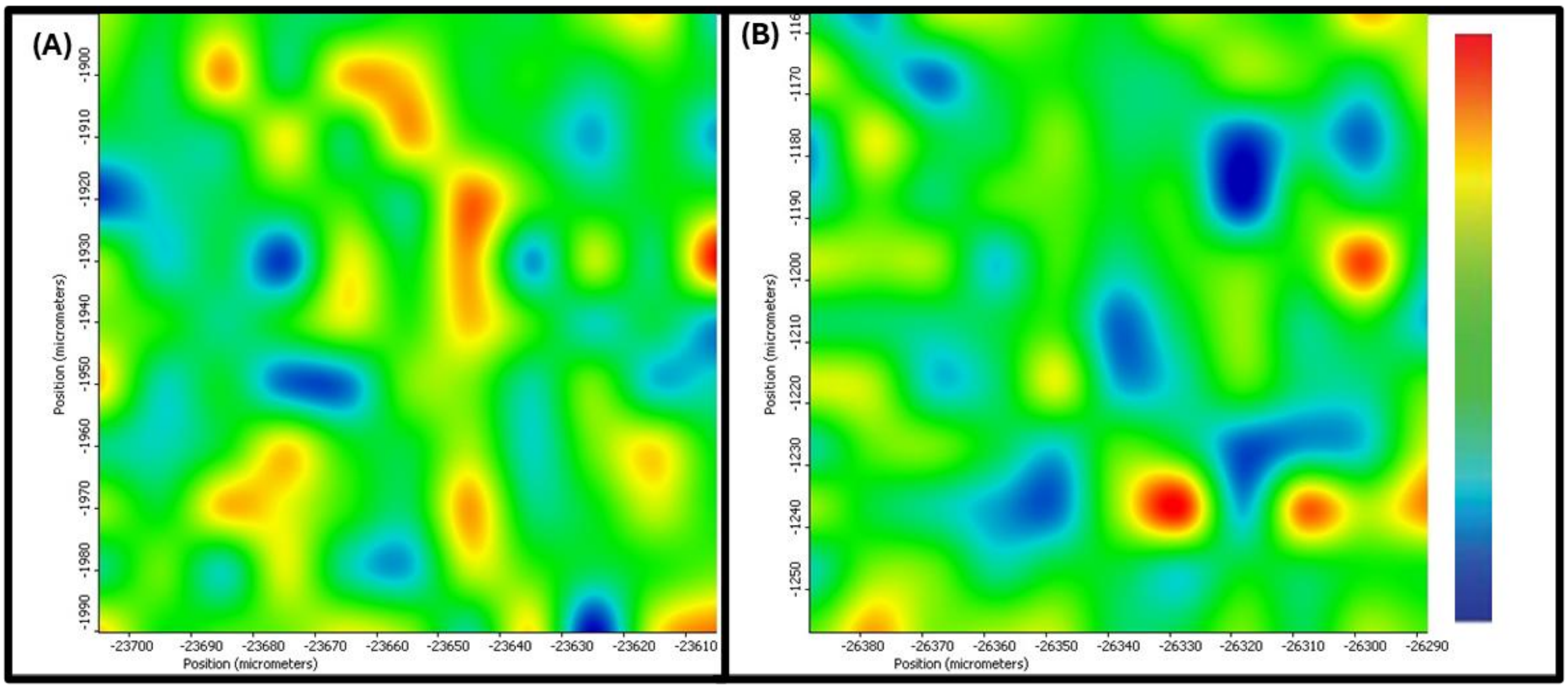

Figure S4. Raman maps of the peak height ratio of the D:G bands for **A)** As-printed and **B)** Activated CB/HDPE AMEP. Red = high D:G ratio, green = balanced structure, blue = low D:G ratio.

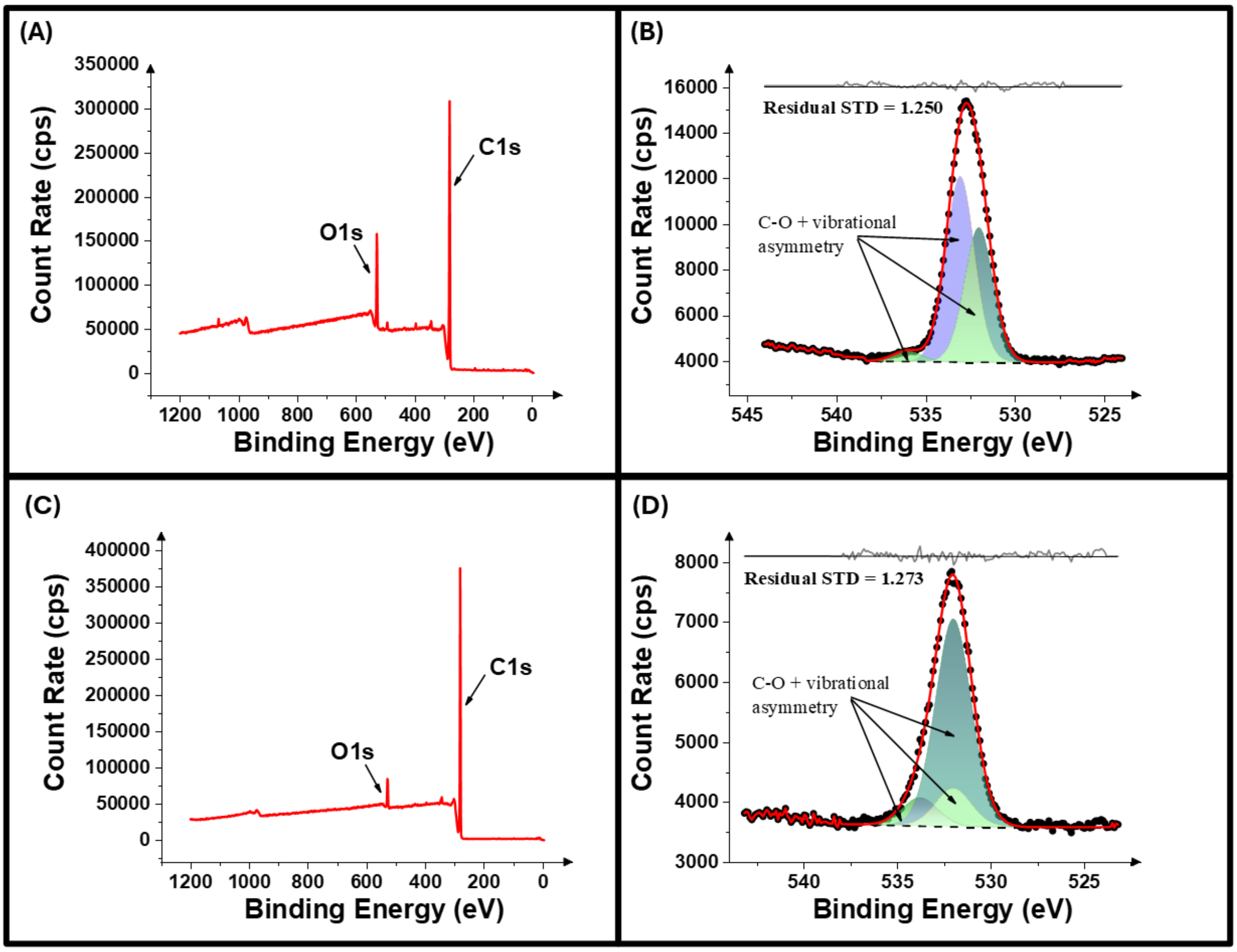

Figure S5. XPS analysis for the as-printed additive manufactured CB/ HDPE electrochemical platforms where **A)** survey plot and **B)** O 1s peak. XPS analysis for the activated additive manufactured CB/ HDPE electrochemical platform where **C)** survey plot and **D)** O 1s peak.

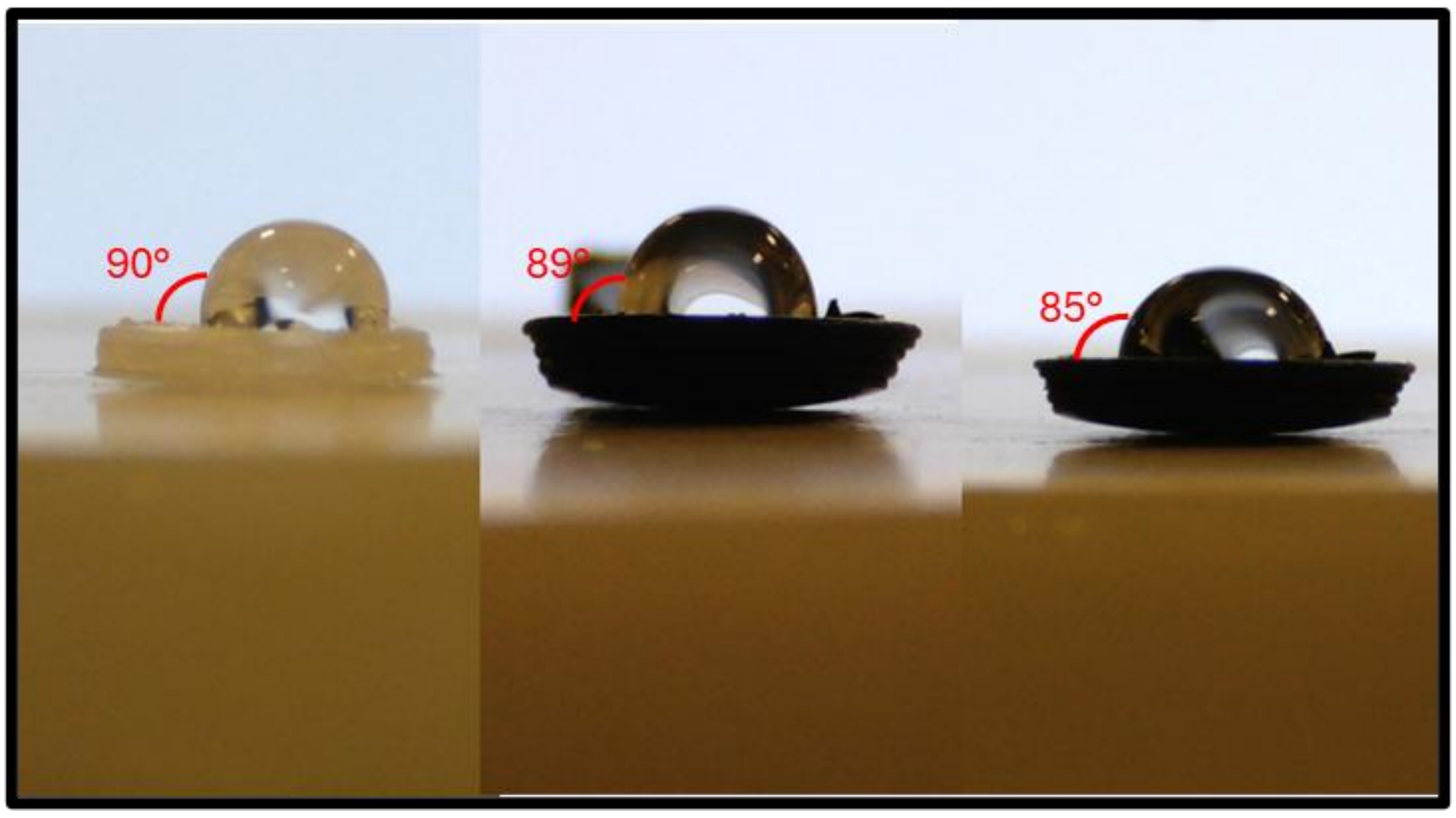

Figure S6. Images of contact angle experiment for the additive manufactured HDPE electrochemical platform, as-printed additive manufactured CB/ HDPE electrochemical platform and activated additive manufactured CB/ HDPE electrochemical platform.

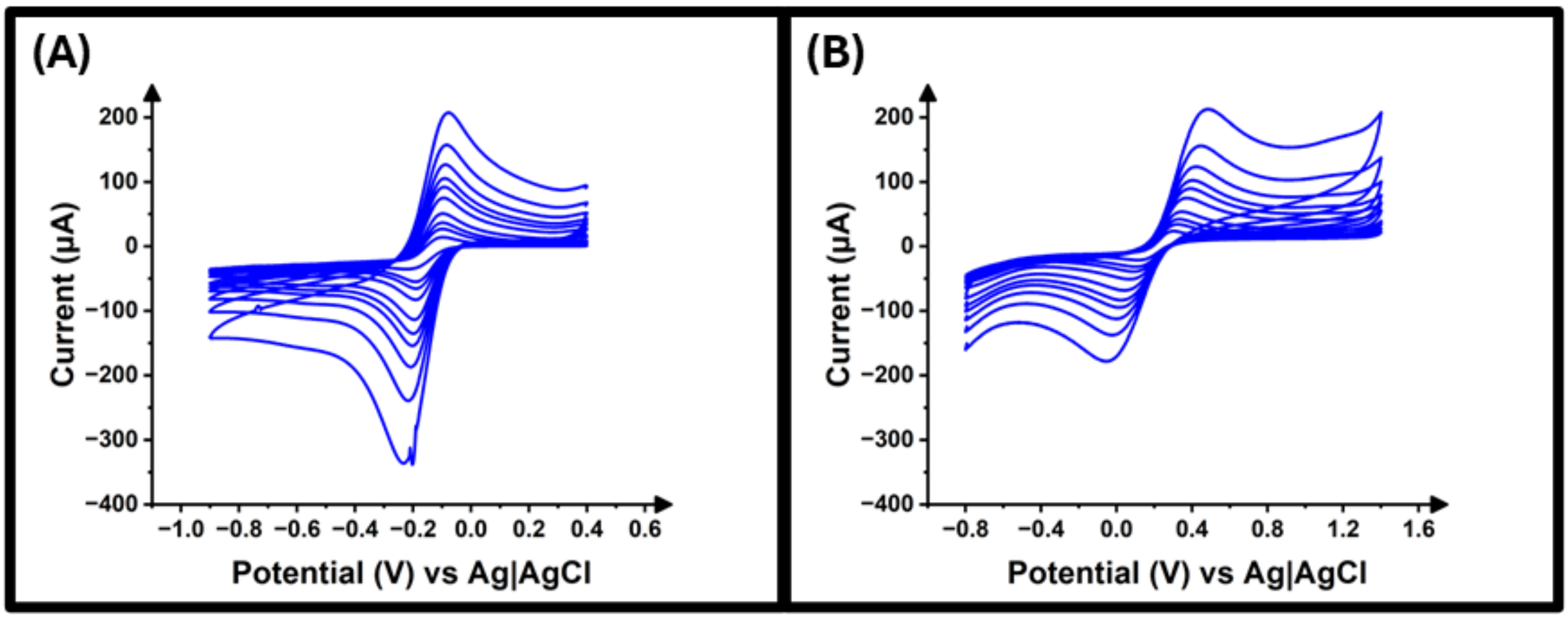

Figure S7. A) Scan rate study (0.005 – 0.5 V s^{-1}) in 1mM $[Ru(NH_3)_6]^{3+}$ / 0.1 M KCl and **B)** scan rate study (0.005 – 0.5 V s^{-1}) in 1mM $[Fe(CN)_6]^{4-}$ / 0.1 M KCl) performed on an activated additive manufactured CB/ HDPE electrochemical platform.

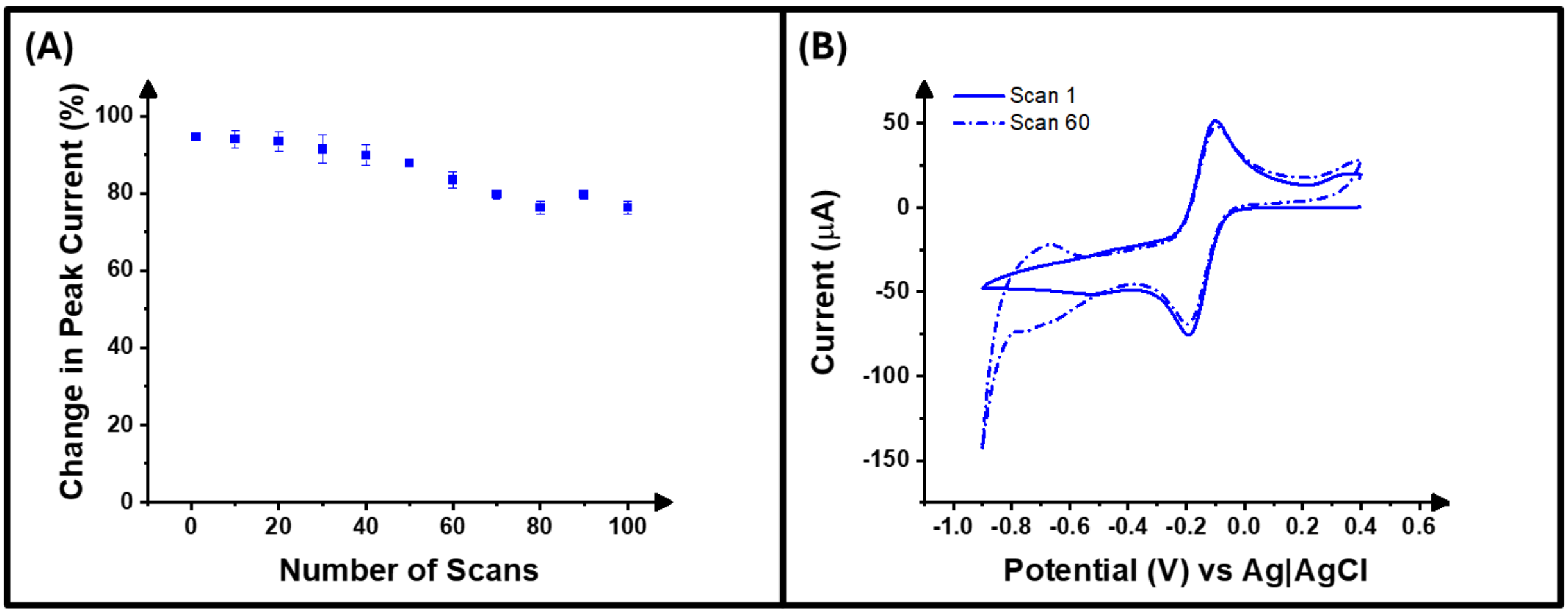

Figure S8. A) Performance of additive manufactured CB/ HDPE electrochemical platform across 100 scans of cyclic voltammetry (0.025 V s^{-1}) in 1 mM $[Ru(NH_3)_6]^{3+}$ / 0.1 M KCl. **B)** Comparison of cyclic voltammograms after 1 scan and 60 scans at 0.025 V s^{-1} in 1 mM $[Ru(NH_3)_6]^{3+}$ / 0.1 M KCl.

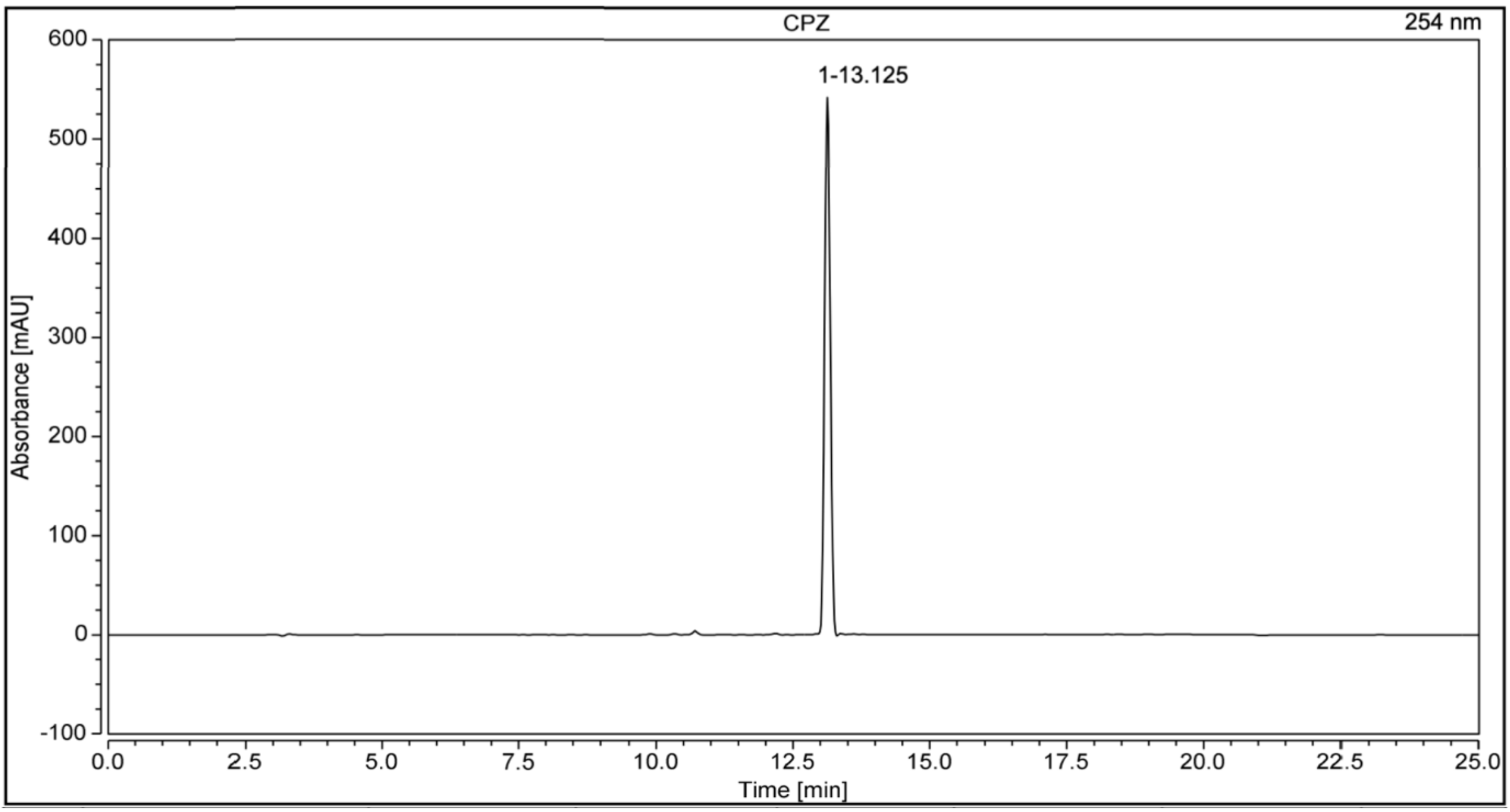

No.	Peak Name	Retention Time	Area mAU*min	Height mAU	Relative Area %	Relative Height %
**1**	Chlorpromazine	13.125	65.694	542.568	76.15	94.34

Figure S9. Chlorpromazine (CPZ) (3 mg/20 mL acetonitrile; 0.15 mg/mL).

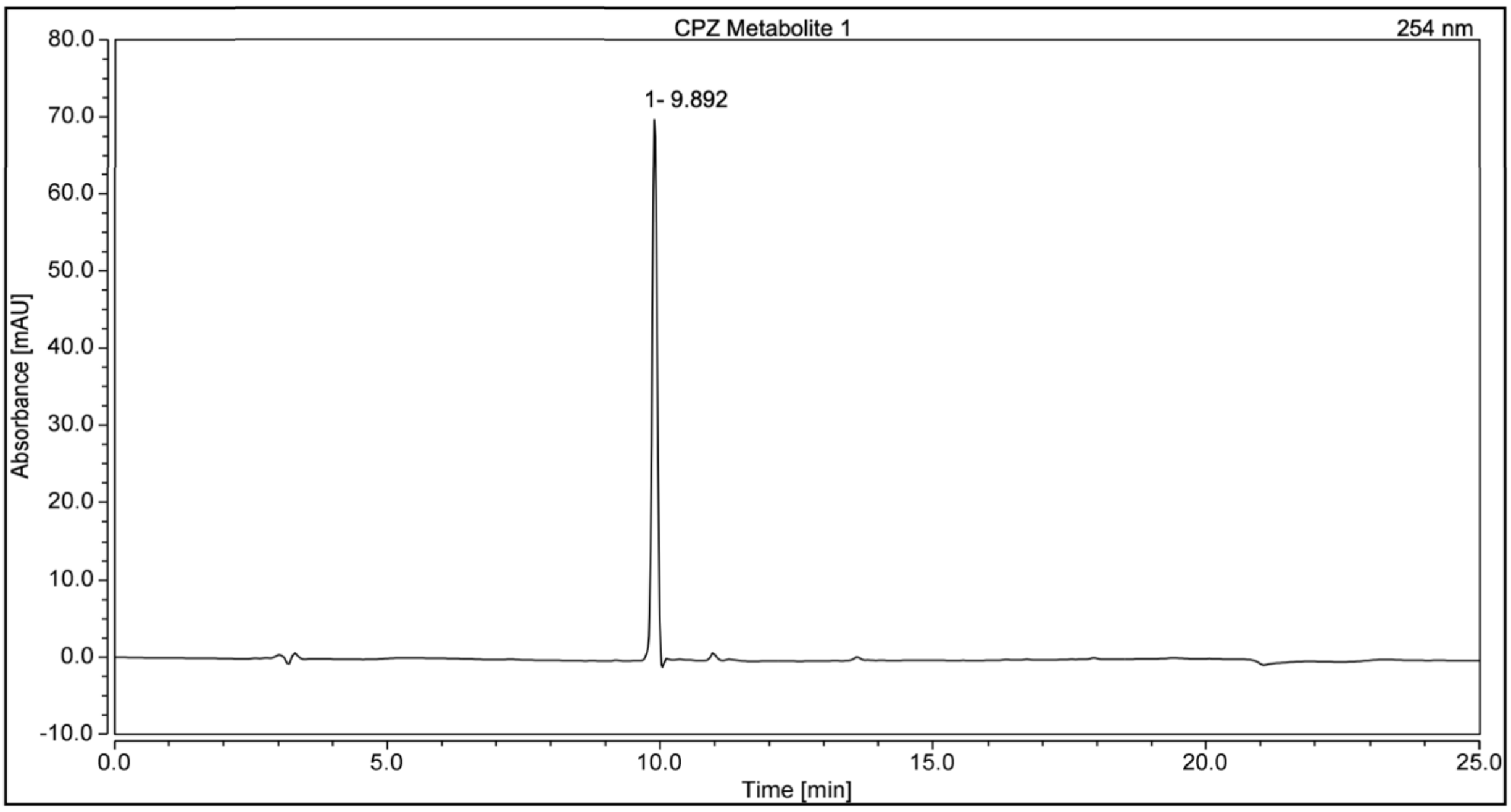

No.	Peak Name	Retention Time	Area mAU*min	Height mAU	Relative Area %	Relative Height %
1	CPZ-sulfoxide	9.892	7.747	70.943	42.17	87.65

Figure S10. CPZ-sulfoxide metabolite.

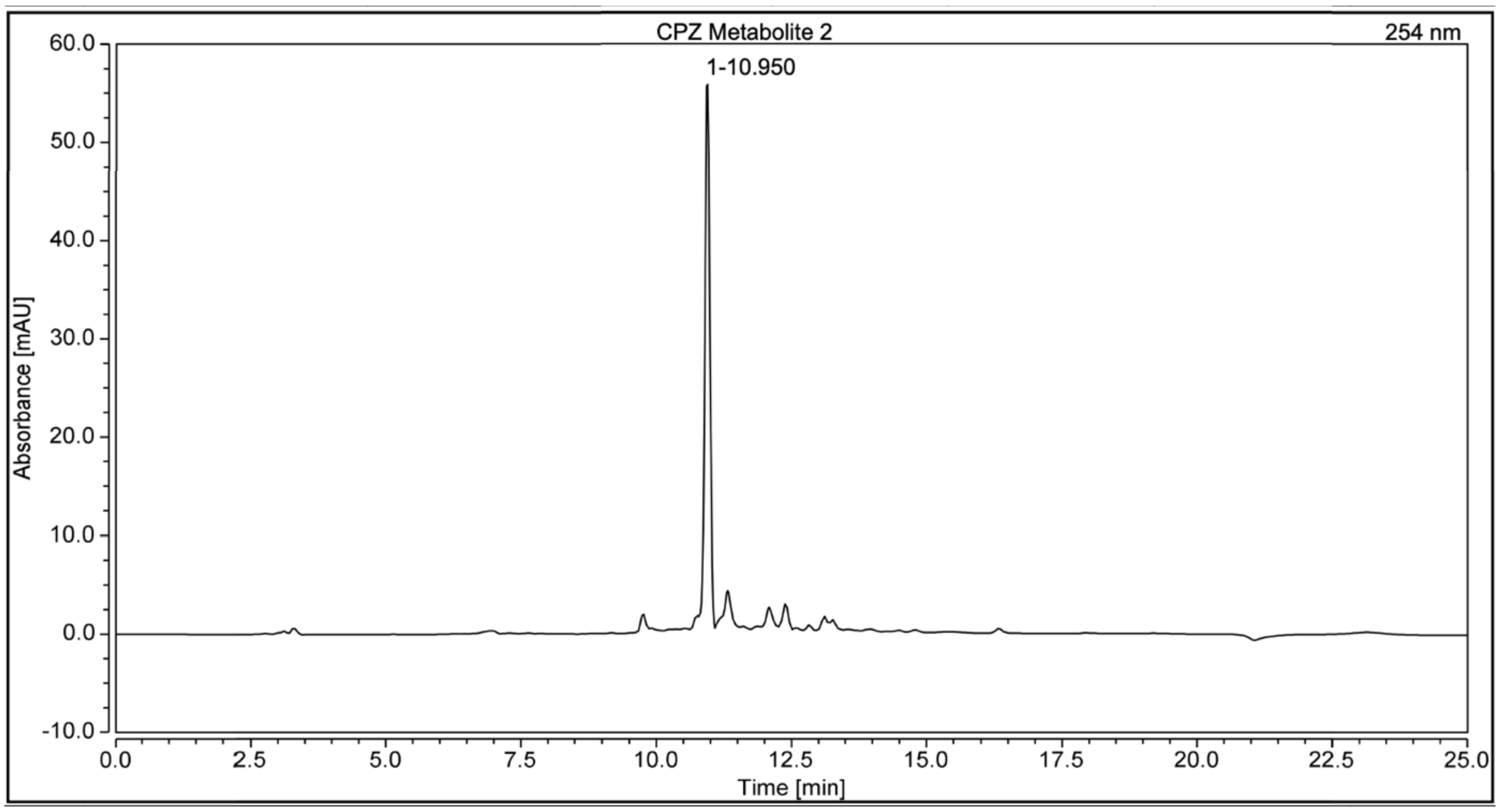

No.	Peak Name	Retention Time	Area mAU*min	Height mAU	Relative Area %	Relative Height %
**1**	CPZ-sulfone	10.950	6.217	55.975	38.03	60.72

Figure S11. CPZ-sulfone metabolite.

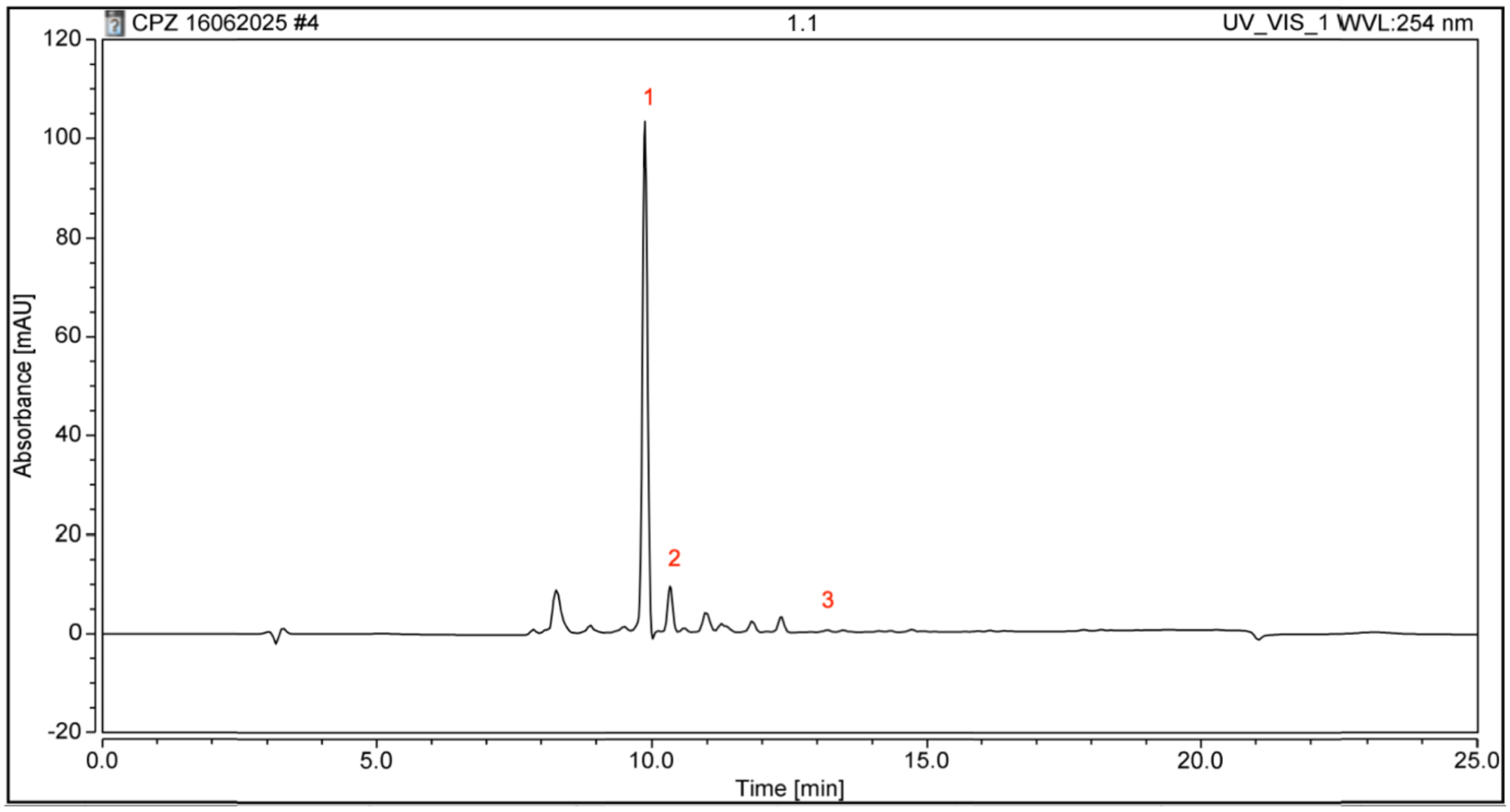

No.	Peak Name	Retention Time	Area mAU*min	Height mAU	Relative Area %	Relative Height %
**1**	CPZ-sulfoxide	9.875	11.457	104.499	28.2	61.48
**2**	CPZ-sulfone	10.325	1.642	10.331	4.04	6.08
**3**	CPZ	13.175	0.190	0.614	0.47	0.36

Figure S12. Entry 3: The first reaction of CB/HDPE additive manufactured electrode without pretreatment.

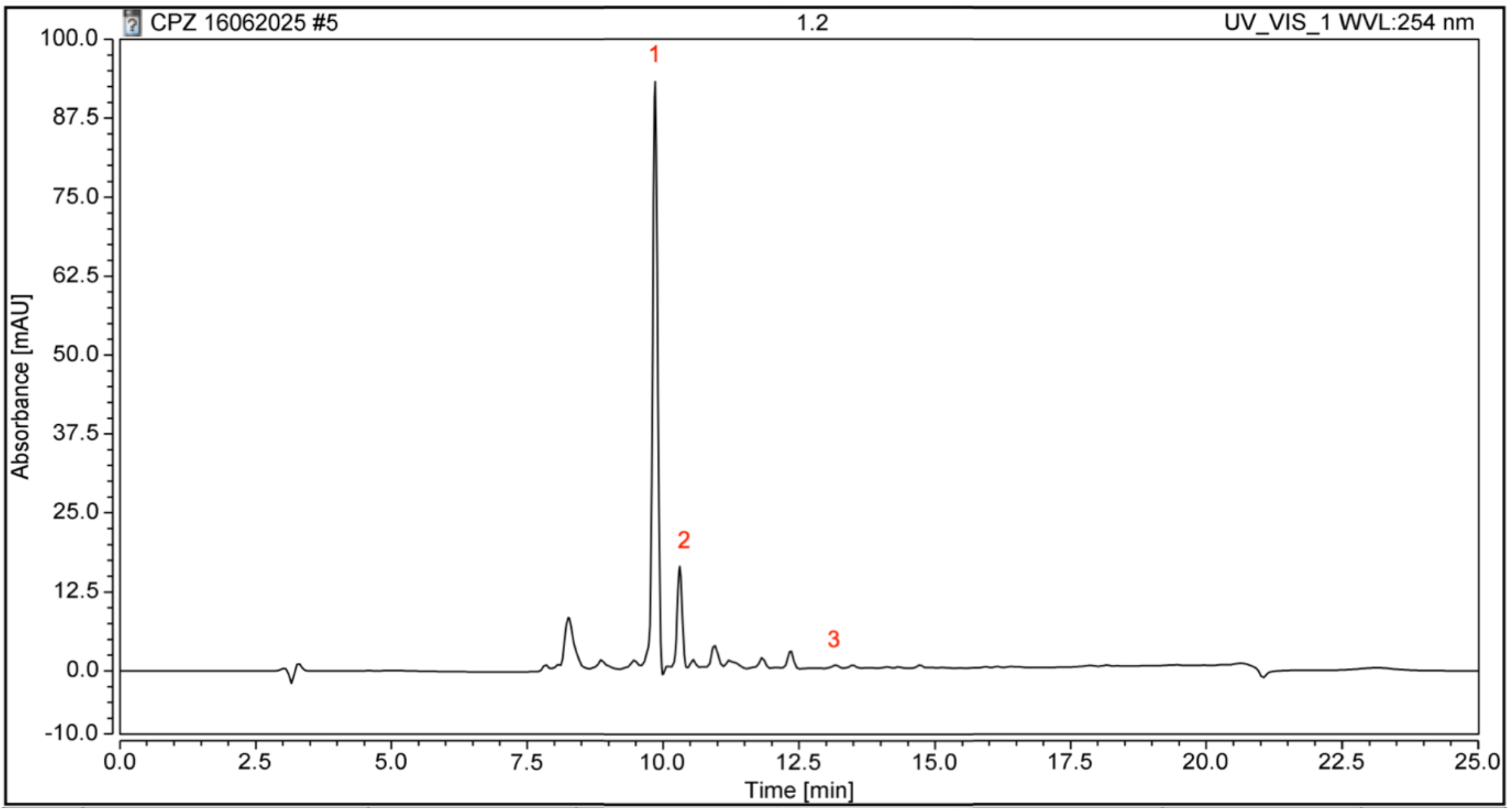

No.	Peak Name	Retention Time	Area mAU*min	Height mAU	Relative Area %	Relative Height %
**1**	CPZ-sulfoxide	9.859	10.903	94.923	19.52	47.82
**2**	CPZ-sulfone	10.309	2.240	18.166	4.01	9.15
**3**	CPZ	13.175	0.735	2.401	1.32	1.21

Figure S13. Entry 4: The second reaction of CB/HDPE additive manufactured electrode without pretreatment.

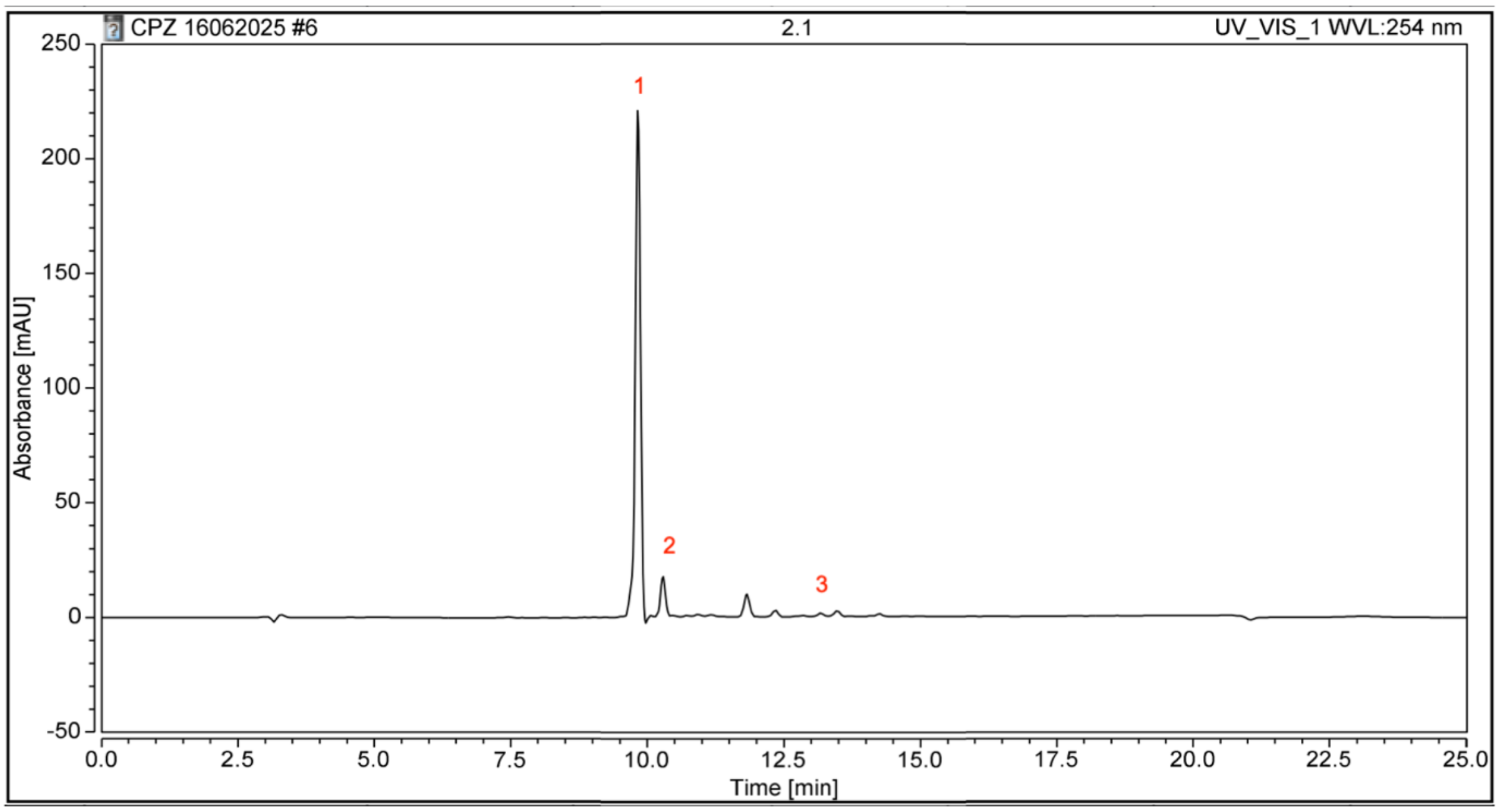

No.	Peak Name	Retention Time	Area mAU*min	Height mAU	Relative Area %	Relative Height %
**1**	CPZ-sulfoxide	9.825	25.474	223.362	33.79	71.47
**2**	CPZ-sulfone	10.292	3.383	20.158	4.49	6.45
**3**	CPZ	13.159	0.164	1.379	0.22	0.44

Figure S14. Entry 5: The first reaction of CB/HDPE additive manufactured electrode with pretreatment.

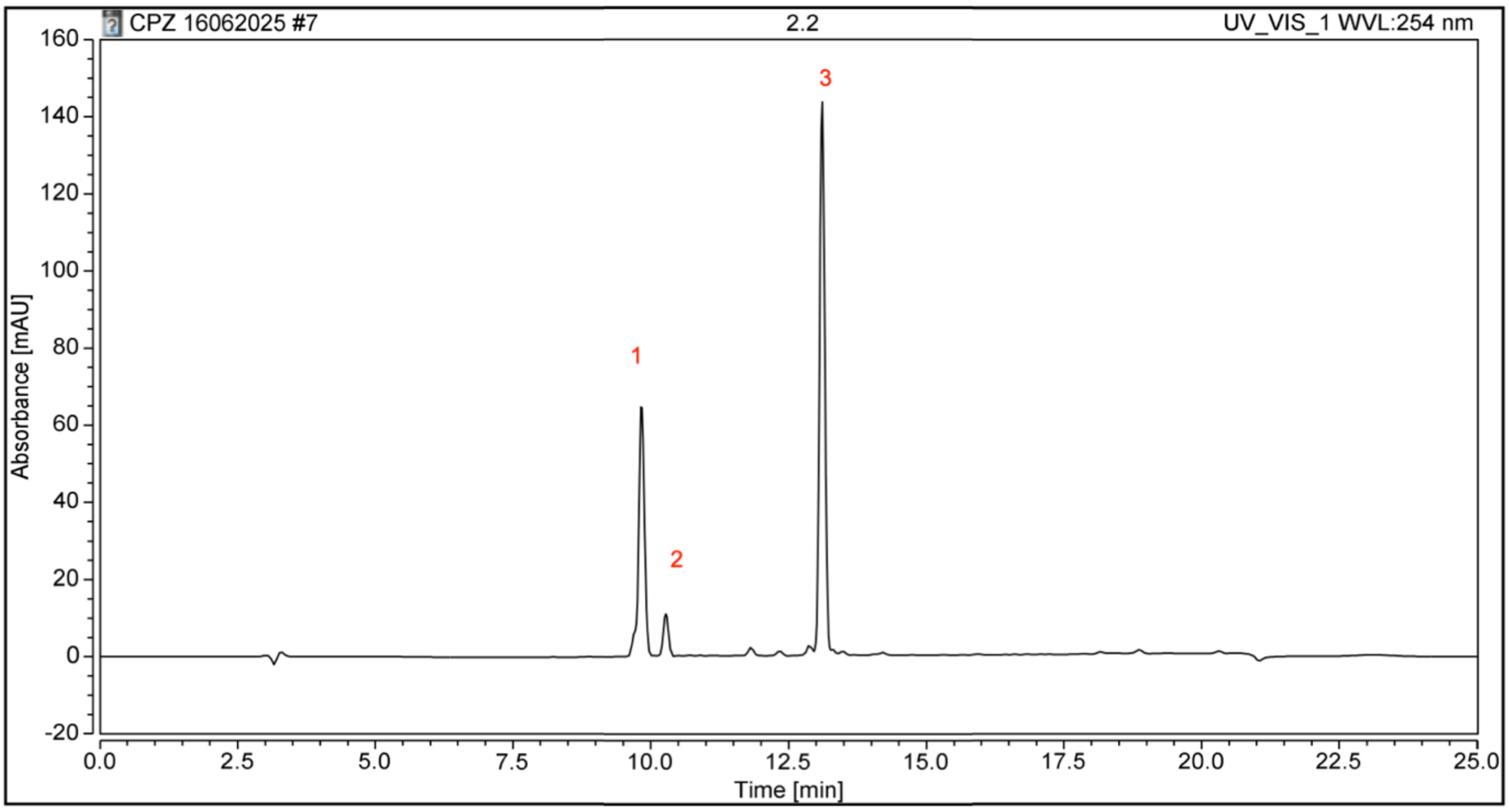

No.	Peak Name	Retention Time	Area mAU*min	Height mAU	Relative Area %	Relative Height %
**1**	CPZ-sulfoxide	9.825	7.735	64.914	17.23	25.25
**2**	CPZ-sulfone	10.275	1.178	11.024	2.62	4.29
**3**	CPZ	13.109	15.845	143.505	35.29	55.83

Figure S15. Entry 6: The second reaction of CB/HDPE additive manufactured electrode with pretreatment.

Compound Characterisation

CPZ-sulfoxide

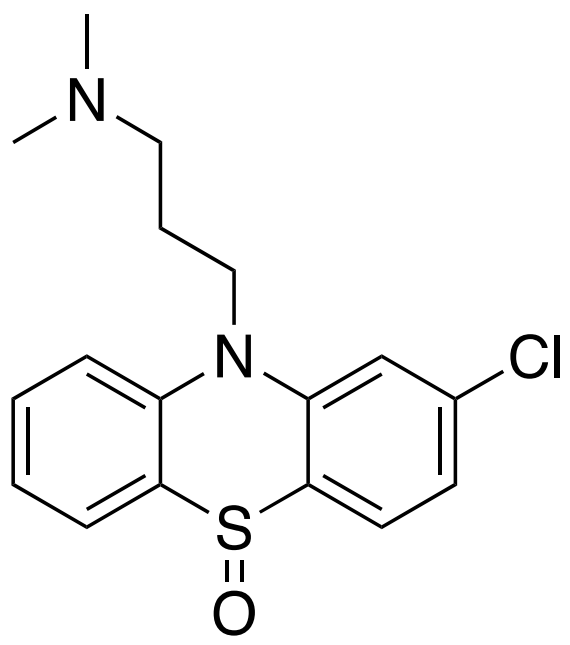

Following the general electrosynthesis procedure CPZ (100 mg, 0.31 mmol), $TBAPF_6$ (608 mg, 1.57 mmol), and the work-up procedures afforded the title compound (33 mg, 0.10 mmol, 32%) as a brown solid. **M.P** 157-161°C. **^{1}H NMR** (400 MHz, DMSO-d_6) δ_H 8.13 – 7.16 (m, 7H), 4.41 (t, J = 7.4 Hz, 2H), 2.70 (td, J = 7.1, 3.0 Hz, 2H), 2.38 (s, 6H), 2.09 – 1.92 (m, 2H).**^{13}C NMR** (101 MHz, DMSO) δ_C 139.1, 137.7, 137.4, 133.2, 132.8, 131.1, 124.6, 123.2, 122.4, 121.7, 116.87, 116.3, 54.7, 44.5, 43.8, 22.7. **LRMS** (ES$^+$) *m/z* 335.10 ([M+H]$^+$, 100%). **HRMS** (EI$^+$) *m/z* $C_{17}H_{19}ClN_2OS$ requires 334.0901, found 334.0904 ([M]$^+$).

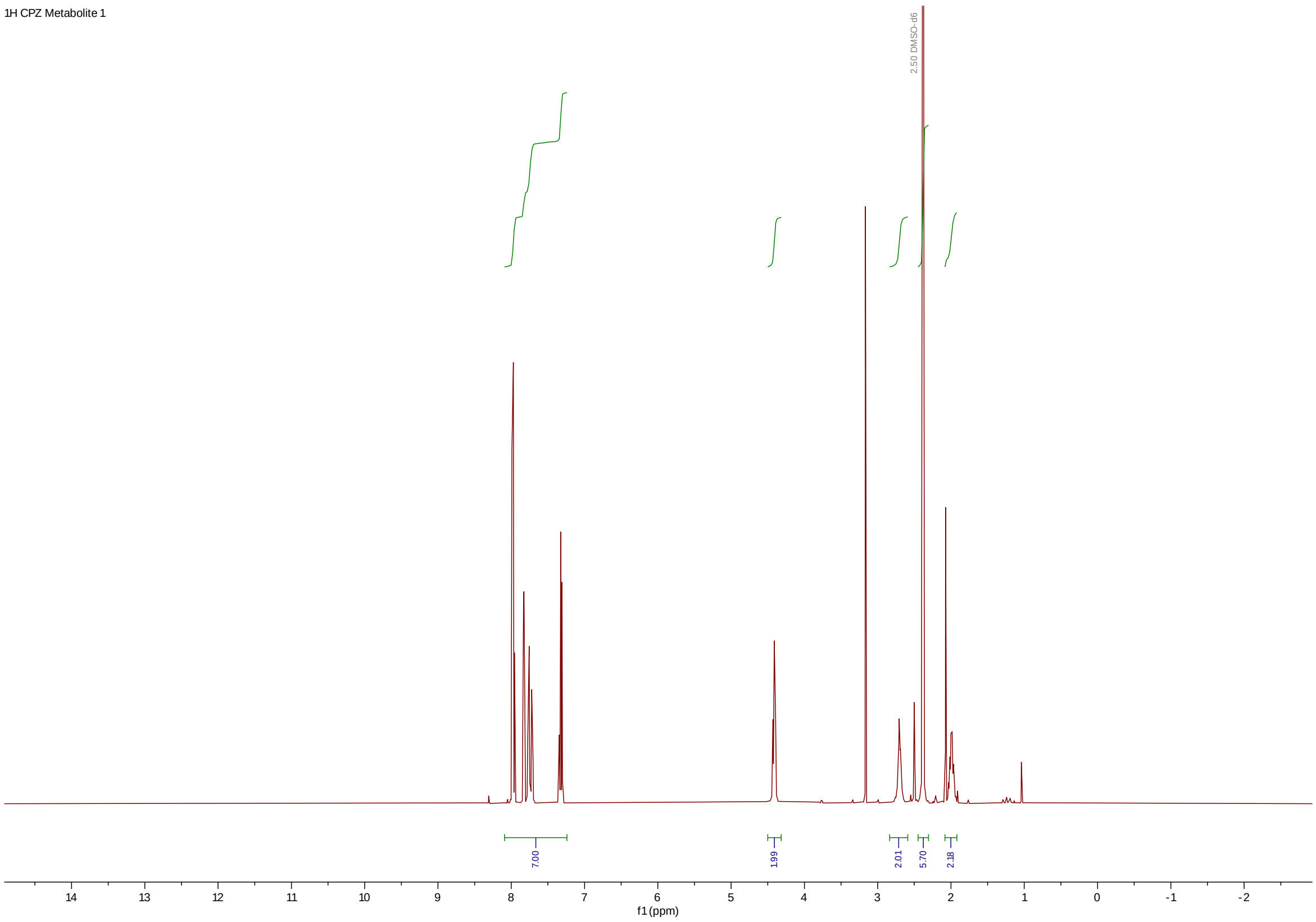

Figure S16: ^{1}H NMR (400 MHz, DMSO-d_6) of CPZ-sulfoxide.

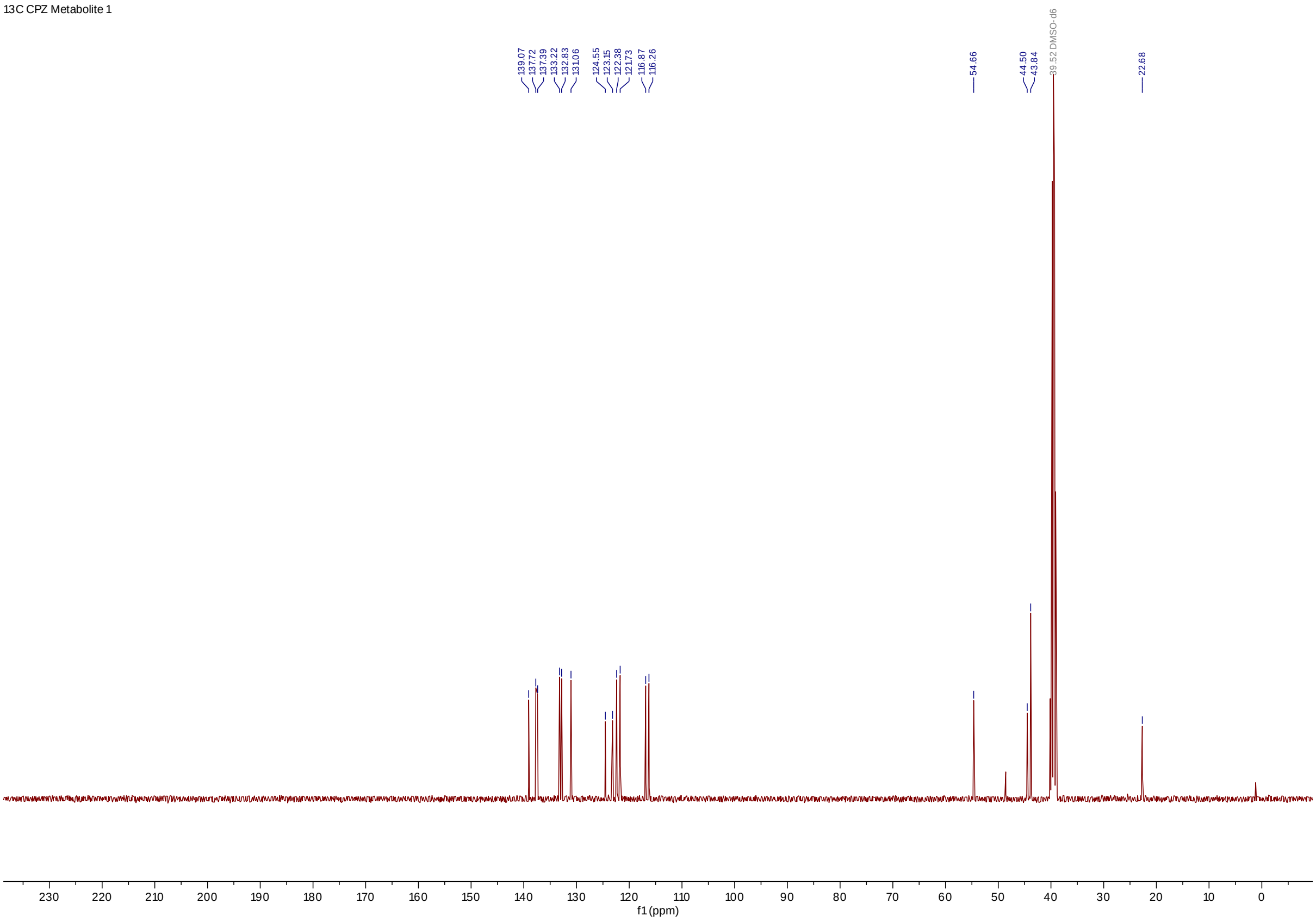

Figure S17: ^{13}C NMR (101 MHz, DMSO-d_6) of CPZ-sulfoxide.

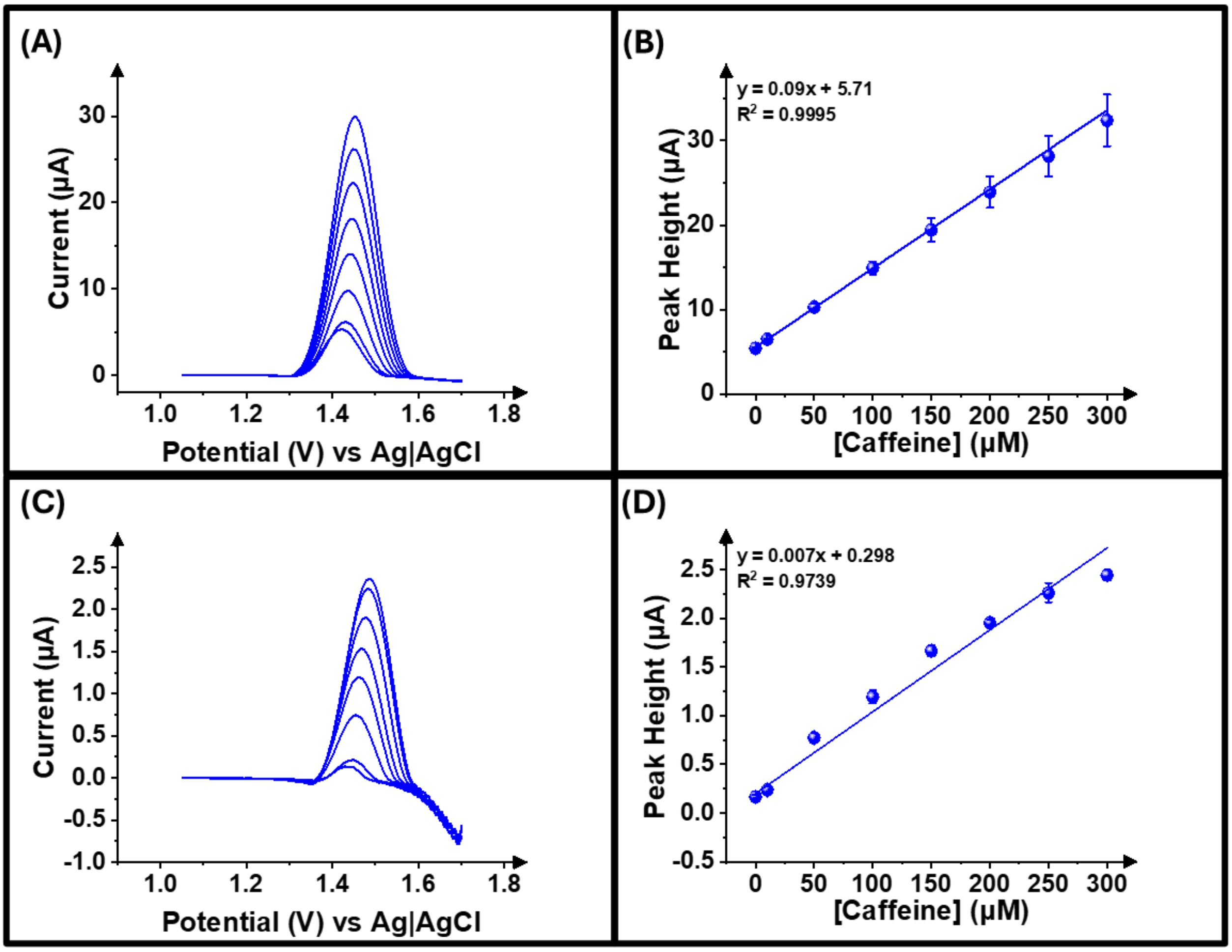

Figure S18. A) Differential pulse voltammetry plot showing response of standard addition of caffeine into Red Bull. **B)** calibration plot for Red Bull. **C)** Differential pulse voltammetry plot showing response of standard addition of caffeine into espresso. **D)** calibration plot for espresso. Differential pulse voltammetry was used with the following parameters: potential range = 0 – 1.7 V, step potential = 0.005 V, modulation amplitude = 0.05 V, modulation time = 0.05 s, interval time = 0.5 s with a pH 5 Buffer. (n = 3).

References

1. Bernalte, E.; Crapnell, R. D.; Messai, O. M.; Banks, C. E., *ChemElectroChem* **2024,** *11* (4), e202300576.
2. Wunderlich, B.; Czornyj, G., *Macromolecules* **1977,** *10* (5), 906-913. DOI 10.1021/ma60059a006.
3. H.J.Bunge, *Texture Analysis in Material science - expansion of orientation distrubutions by spherical harmonics*. **1982**.
4. Tarani, E.; Arvanitidis, I.; Christofilos, D.; Bikiaris, D. N.; Chrissafis, K.; Vourlias, G., *Journal of Materials Science* **2023,** *58* (4), 1621-1639. DOI 10.1007/s10853-022-08125-4.
5. Greczynski, G.; Hultman, L., *Scientific reports* **2021,** *11* (1), 11195.
